# A Virtual Trial to Identify Cardiovascular Biomarkers for Differentiating Diabetic and Hypertensive Kidney Disease

**DOI:** 10.1007/s10439-026-03983-4

**Published:** 2026-01-29

**Authors:** Ning Wang, Steven P Sourbron, Ivan Benemerito, Alberto Marzo

**Affiliations:** 1 INSIGNEO Institute for in silico medicine, University of Sheffield, Sheffield, UK; 2 School of Mechanical, Aerospace and Civil Engineering, University of Sheffield, Sheffield, UK; 3 School of Medicine and Population Health, University of Sheffield, Sheffield, UK

**Keywords:** Diabetic kidney disease, Hypertensive kidney disease, Computational fluid dynamic, Biomarker, Logistic regression model

## Abstract

**Purpose:**

A diagnostic challenge in the management of chronic kidney disease (CKD) is distinguishing diabetic kidney disease (DKD) from hypertensive kidney disease (HKD) in patients with coexisting diabetes mellitus (DM) and hypertension (HTN), because accurate diagnosis often depends on renal biopsy as a reference standard. This study proposes a modeling approach to identify cardiovascular biomarkers for differentiating DKD from HKD.

**Methods:**

An existing whole-body circulation model of the vascular tree was extended with a detailed renal circulation network to predict biomarkers measured at different locations. The model parameterized sex, age, and disease factors and was used to conduct virtual clinical trials that identified individual and combined biomarkers for DKD-HKD differentiation. Biomarkers were identified with univariate and multivariate analysis and characterized with the area under the receiver operating characteristic curve (AUC).

**Results:**

Results show that the strongest individual biomarker that is commonly used in clinical practice is pulsatility index (PI) measured in the main renal artery, with an AUC of 0.87. Among all evaluated two-biomarker combinations, PI and resistive index (RI) measured in the same artery achieved the highest classification performance (AUC 0.94). In comparison, the highest performance among three-biomarker combinations (AUC 0.96) is achieved by mean blood flow rate, systolic blood flow rate, and diastolic flow rate.

**Conclusion:**

This modeling work suggests that cardiovascular biomarkers can assist in differentiating DKD and HKD, and proposes specific hypotheses that form a strong rationale for targeted clinical trials. If confirmed, these methods could enable non-invasive assessment of renal vascular alterations associated with DKD and HKD, reducing reliance on kidney biopsies for diagnostic evaluation.

**Supplementary Information:**

The online version contains supplementary material available at 10.1007/s10439-026-03983-4.

## Introduction

CKD is a global health condition characterized by a progressive decline in kidney function, with more than 844 million people affected by CKD worldwide since 2017 [1]. CKD demonstrates sex- and age-specific differences in the United States, with a prevalence of 15% in females and 11% in males [2], and a prevalence of 52% in individuals older than 40 years old (yo), compared to 9% in individuals between the ages of 20 and 39 [3].

The rising prevalence of DM and HTN significantly contributes to the global increase in CKD cases. Research indicates that CKD prevalence ranges from 19% to 66% among diabetic patients and from 30% to 51% among hypertensive patients [4]. DKD is a microvascular complication of DM, where chronic hyperglycemia induces a cascade of metabolic and hemodynamic disturbances [5], while HKD is a macrovascular complication resulting from the effects of chronically elevated blood pressure on the renal vasculature [6, 7]. DKD and HKD may benefit from targeted management, but this can only be considered if the cause of CKD is known. Unfortunately, since DM and HTN are common comorbidities, and DKD and HKD share similar symptoms, this is often only possible by invasive biopsy. Since this is not considered a viable option in early disease stages, most patients are not able to benefit from targeted management. Specific, non-invasive diagnostic measurements are urgently needed to allow earlier and more effective management of CKD in patients with DM and HTN.

We hypothesize that systemic and microvascular alterations caused by DKD and HKD lead to characteristic signatures in hemodynamic biomarkers that can be measured with flow-sensitive acquisition protocols, such as Doppler ultrasound (US) and phase-contrast magnetic resonance imaging (PC-MRI). In patients with DKD, chronic hyperglycemia causes vasodilation of the afferent arterioles (reducing vascular resistance) and vasoconstriction of the efferent arterioles (increasing vascular resistance). These hemodynamic perturbations increase glomerular blood flow, resulting in prolonged hyperfiltration that ultimately leads to nephron loss [8]. Conversely, in patients with HKD, pathological overactivation of the renin–angiotensin–aldosterone system triggers sustained vasoconstriction in both afferent and efferent arterioles (increasing vascular resistance) [9]. Unlike the glomerular hyperfiltration characteristic of DKD, HKD manifests as vascular remodeling, evidenced by structural thickening and fibrotic stiffening of renal arterial walls. It appears plausible that these distinct micro- and macrovascular alterations of DKD and HKD lead to distinct patterns in flow-sensitive Doppler US and PC-MRI, but the relationship is complex and cannot be studied in humans by experimental means. Unfortunately, animal models poorly represent complex human diseases and do not properly replicate the experimental conditions of human imaging.

In recent decades, evidence has been mounting that reduced-order models of the cardiovascular system can effectively simulate pathological effects on pressure, velocity, and flow waves. These models have demonstrated potential in identifying diagnostic biomarkers, as seen in conditions such as pulmonary HTN [10], cerebral vasospasm [11], or coronary artery disease [12]. This strategy complements traditional machine learning methodologies by enabling the quantitative analysis of biomarker input data, thereby facilitating the detection of subtle and nonlinear patterns that are often imperceptible through conventional observational techniques. However, current models are inadequate for the detailed study of CKD. Most prior studies target the main renal artery or the renal microcirculation in isolation [13–16], leaving a gap in models that concurrently represent pulse-wave propagation in the proximal renal vasculature and disease pathophysiology. Therefore, a higher-resolution framework is required to capture interactions between the proximal renal vasculature and the microcirculation that characterize early-stage DKD and HKD.

This study advances an established renal model with three novel contributions compared with existing studies [17]. The renal circulation is extended to the arcuate arteries for a more detailed representation of the proximal vasculature, and the renal microcirculation is modeled as a detailed equivalent electrical circuit represented by a lumped-parameter resistor–capacitor–resistor (R-C-R) model. This representation enables mechanistic estimates of glomerular filtration rate (GFR). In addition to age-specific factors, vascular properties are parameterized by biological sex, providing more detail on interindividual variability than prior studies [17–19]. Furthermore, a more clinically relevant scenario was modeled, in which the pathophysiology of DKD and HKD was parameterized based on coexisting DM and HTN to support biomarker identification. Collectively, these enhancements deliver a virtual population-level framework for quantifying biomarker discrimination between DKD and HKD.

This study aimed to identify and evaluate individual and composite biomarkers for the early differentiation of DKD and HKD using a multidimensional (1D-0D) model combined with a logistic regression-based machine learning approach, as detailed in Section "Univariate and multivariate logistic regression model" of the Methodology. This framework enables modeling assessment of disease-specific hemodynamic alterations between DKD and HKD pathological conditions. The findings provide model-level insights that may inform clinical trial protocols and support the development of more targeted, accessible, and early-stage strategies for distinguishing DKD from HKD, which are often clinically indistinguishable in their early stages.

## Methodology

An existing vascular network model was extended to incorporate renal microcirculation, enabling the simulation of disease-specific hemodynamic responses. The model was parameterized to account for sex-, age-, and disease-specific variations related to coexisting DM and HTN, DKD, and HKD. In the following sections, the term “virtual healthy controls” denoted disease-free simulated subjects generated under age- and sex-specific parameter distributions. The term “virtual patients” denoted simulated subjects instantiated with disease-specific parameterizations (DM + HTN, DKD, or HKD). Using this framework, virtual clinical trials were conducted to identify candidate biomarkers that distinguish DKD from HKD and to assess their diagnostic accuracy.

### Systemic and Renal Circulation Model

**Fig. 1:**
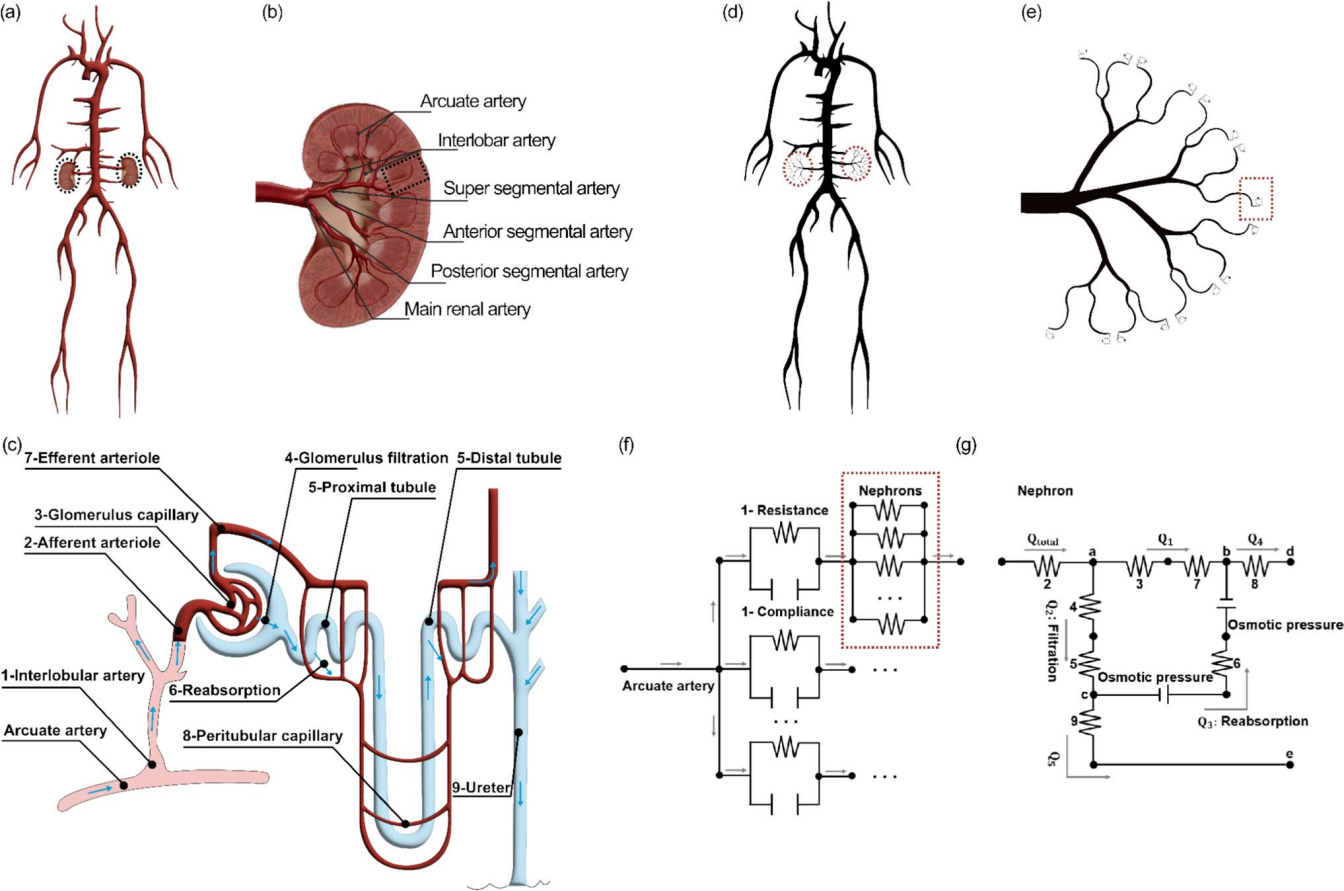
Illustration of the systemic whole-body circulation and renal circulation, including both anatomical structures and model representations, (**a**) anatomical systemic circulation network including left and right kidneys, (**b**) renal vasculature from the main renal artery to the arcuate artery, (**c**) renal microcirculation network, (**d**) multidimensional systemic circulation model incorporating left and right renal circulation networks, (**e**) renal vasculature represented as 1D blood vessels, with microcirculation downstream of the arcuate artery represented using a lumped-parameter R-C-R model, (**f**) coupling scheme between the 1D arcuate artery and the lumped-parameter R-C-R model, (**g**) constituent components of the lumped-parameter R-C-R model within a single nephron

The vascular network in this study was illustrated in Fig 1. It consisted of three components: (1) a systemic circulatory network, (2) bilateral 1D renal arterial networks (right and left), (3) 0D renal microcirculatory networks. The dimensional values of the model for the age group of 20–29 yo were presented in the supplementary material.

#### Systemic Circulatory Model

The systemic circulation network followed a previous publication, comprising 75 blood vessels represented using a 1D modeling approach, and 29 outlet boundary conditions [17]. They were represented using the lumped-parameter R-C-R models to represent the peripheral blood vessels. The inlet boundary condition was defined as the blood flow rate in the ascending aorta.

#### 1D Renal Circulatory Model

The newly developed renal circulatory model was derived from a previously published renal architecture consisting of 1 main renal artery, 5 segmental arteries, and 10 interlobar arteries per kidney [17]. The extension involved the addition of bifurcations at the peripheral termini of each interlobar artery, thereby forming two arcuate arteries per branch, resulting in a total of 20 arcuate arteries per renal circulation, as shown in Fig. 1e. These renal arteries were represented using a 1D modeling approach, with mechanical properties sourced from previously published literature [20–23], and detailed in the supplementary material.

#### 0D Renal Microcirculatory Model

Downstream vasculature of each arcuate renal artery was the renal microcirculation, comprising interlobular arteries, afferent arterioles, glomerular capillaries, efferent arterioles, renal tubules, and peritubular capillaries. The overall resistance of the renal microcirculation, including both vascular components and critical renal functions involving glomerular filtration and tubular reabsorption, was represented using the lumped-parameter R-C-R model, as shown in Fig. 1f and 1g.

To calculate the resistance of the renal microcirculation (PVR downstream of each arcuate renal artery), the following assumptions were applied. Firstly, interlobular arteries were modeled in a uniform and parallel configuration, with 650 of these branching at the peripheral terminus of each arcuate artery [23]. Each interlobular artery subsequently branches at its peripheral terminus into 60 glomeruli in a parallel configuration [24], as shown in Fig. 1f. Secondly, hemodynamics was homogeneous among the components within the renal microcirculation, manifesting uniform resistive properties and invariant pressure-flow relationships. The total number of each component within the lumped-parameter R-C-R model was summarized in Table 1.

**Table 1:** Mechanical properties and estimated number of interlobular arterioles, afferent and efferent arterioles, and renal tubules

Parameters	Label	Number	Viscosity [Pa·s]	Length [µm]	Radius [µm]	Resistance [Pa·s/m^3^]
Interlobular	1	13,000	4.00 × 10^−3^	370	22.90	1.37 × 10^−13^
Afferent	2	784,909	4.00 × 10^−3^	112	10.70	8.71 × 10^−13^
Glomerular capillary	3	N/A	N/A	N/A	N/A	3.92 × 10^−12^
Glomerular filter	4	N/A	N/A	N/A	N/A	3.35 × 10^−14^
Renal tubule	5	784,909	2.00 × 10^−3^	18,000	16.40	1.27 × 10^−15^
Reabsorption	6	N/A	N/A	N/A	N/A	3.31 × 10^−14^
Efferent	7	784,909	2.00 × 10^−3^	120	7.97	1.52 × 10^−14^
Peritubular capillary	8	N/A	N/A	N/A	N/A	3.60 × 10^−13^
Ureter	9	N/A	N/A	N/A	N/A	7.96 × 10^−15^

Number of interlobular arteries [23], afferent arterioles [24], blood viscosity [29], plasma viscosity [30], length of interlobular artery [23], afferent arteriole [31], renal tubule [32, 33], and efferent arteriole [34]. Lumen radius of interlobular artery [34], afferent arteriole [31], renal tubule [35], and efferent arteriole [31]

**Fig. 2:**
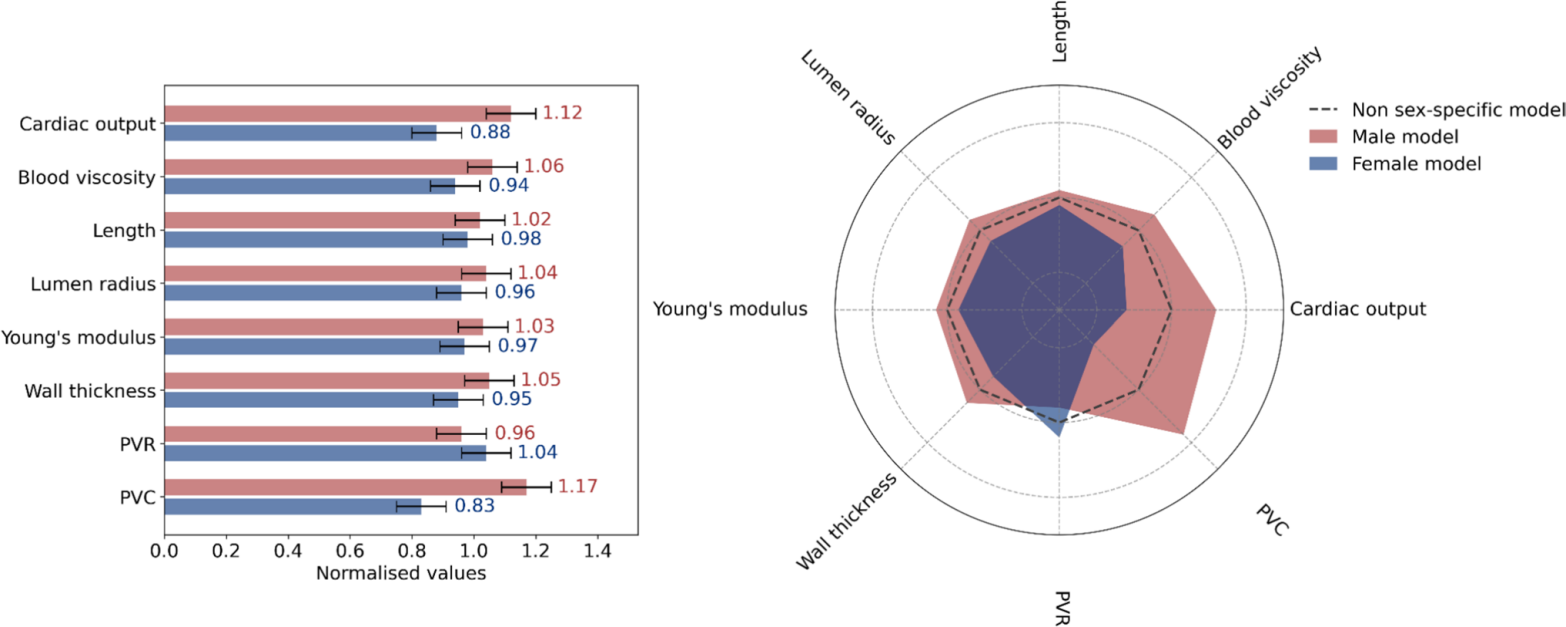
Normalized mechanical properties between males and females, while the PVR ratio in renal circulation is 0.85 in males and 1.15 in females

The Hagen–Poiseuille equation was employed to determine the vascular resistance of interlobular artery, afferent arteriole, efferent arteriole, and renal tubule within the lumped-parameter R-C-R model (Fig. 2g), considering the low pulsatility within renal microcirculation [25]. Furthermore, the resistances of the glomerular capillary, glomerular filter, peritubular capillary, and reabsorption were calculated based on their ratios to the resistance of the afferent arteriole [26]. The vascular resistance for each component within the lumped-parameter R-C-R model is summarized in Table 1.

To calculate the total vascular resistance downstream of the arcuate artery, two steps were followed. Firstly, the vascular resistance of a nephron was calculated as 4.40 × 10^12^ Pa·s/m^3^ by summing the resistance of the afferent arteriole and the cumulative resistance of its downstream microvasculature arranged in a series configuration. The vascular resistance downstream of the afferent arteriole, as shown in Fig. 1f, was determined using nodal analysis based on Kirchhoff’s law. Secondly, the total vascular resistance downstream of an arcuate artery was calculated as 2.80 × 10^10^ Pa·s/m^3^ based on two levels of parallel configuration. In the first level, 60 nephrons were arranged in parallel to an interlobular artery. In the second level, 650 interlobular arteries (each containing 60 nephrons) were arranged in parallel to an arcuate artery. The resistance of an interlobular artery employed in this calculation was reported in Table 1. Full details of the calculations are presented in the supplementary material.

Furthermore, the PVC downstream of an arcuate was determined by 2.53 × 10^−11^ m^3^/Pa [17, 27]. The GFR for each virtual healthy control and patient was calculated as the ratio of the blood flow rate filtered by the glomerulus to the total blood flow rate entering the glomerulus within the renal microcirculation. To support these numerical computations, this study employed an open-source software openBF to compute blood flow rate, velocity, pressure, and pulse-wave velocity (PWV) in all vessels of the network over a complete cardiac cycle [28].

### Sex, age, and pathophysiology parameterization

This section incorporated variations in sex, age, coexisting systemic DM and HTN, DKD, and HKD into the vascular network model developed in this study.

#### Sex- and age-specific parametrization

Sex and age variations affect several mechanical parameters, including blood viscosity, cardiac output (CO), length, lumen radius, Young’s modulus, wall thickness, PVR, and PVC. To parameterize a sex-specific model, each mechanical property was normalized through dividing the respective CO, blood viscosity, and mechanical values for males and females by their corresponding mean values, based on multiple sources in the literature [36, 37]. These normalized values were subsequently multiplied by the corresponding properties of the newly developed non-sex-specific model, as shown in Fig. 2, ensuring that the mechanical properties represent the variants of males and females.

Regarding the age model, the variations in the aforementioned parameters were assumed to apply equally to both males and females throughout the aging process, with each assumed to follow a Gaussian distribution. A validated age-specific model was subsequently employed to simulate the aging process in both male and female models [17–19], across six distinct age groups ranging from 20 to 79 years.

#### Systemic DM and HTN Parametrization

Systemic vascular damage resulting from the combined pathophysiological effects of DM and HTN (DM + HTN) was modeled prior to the onset of kidney disease. This systemic vascular damage affected the lumen radius [17, 38], Young’s modulus [39], and wall thickness [40] of the 1D blood vessel model. The HTN-induced vascular constriction effect on peripheral blood vessels was further quantified as a 17% increase in PVR and a 23% reduction in PVC [41, 42], alongside a 17% decrease in CO relative to healthy individuals [43, 44]. Additional quantitative data supporting the parameterization of the DM + HTN model are presented in Table 2.

**Table 2:** Normalized mechanical parameters derived from the healthy model for DM + HTN, DM + HTN + DKD, and DM + HTN + HKD models, respectively

Parameters	DM + HTN	DM + HTN + DKD	DM + HTN + HKD
	Normalized	Normalized	Normalized
GFR [ml/min]	93 (10)	84 (8)	85 (8)
Cardiac output	0.83	0.83	0.83
Blood viscosity	1.20	1.20	1.20
Systemic circulation			
Length	1.00	1.00	1.00
Lumen radius	0.97	0.97	0.97
Young’s modulus	1.25	1.25	1.25
Wall thickness	1.12	1.12	1.12
PVR	1.17	1.17	1.17
PVC	0.77	0.77	0.77
Renal circulation			
Length	1.00	1.00	1.00
Lumen radius	0.97	0.97	0.95
Young’s modulus	1.25	1.25	1.40
Wall thickness	1.12	1.12	1.23
PVR	1.17	1.35	1.29
PVC	0.77	0.77	0.77

#### DKD and HKD Parameterization

DKD was classified as a microvascular disease, with renal microcirculation being a primary target for injury. DKD model quantified a 7% constricted lumen diameter of efferent arterioles resulting from hyperfiltration [45], accompanied by a 10% dilated lumen diameter of afferent arterioles, while maintaining other components in the lumped-parameter R-C-R model unchanged. These alterations can decrease the PVR downstream of the arcuate artery by 1%. In addition, a reduction in the total number of glomeruli was known as a consequence of prolonged hyperfiltration-induced overload [46]. A 57% reduction in the number of glomeruli within the renal microcirculation was modeled for DKD patients to ensure consistent GFR between DKD and HKD patients. This adjustment leads to an overall 35% increase in PVR compared with the healthy baseline, resulting in the mean GFR of both virtual patients with DKD and HKD remaining within the same early stage (stage 2) of CKD.

HKD was characterized by proximal renal vascular damage resulting from systemic HTN, which was driven by constriction of peripheral blood vessels. Prolonged high blood pressure can induce structural changes within the renal vasculature, including hypertrophy of the vascular smooth muscle, which led to thickening of the vessel wall and a reduction in elastin, compromising the elasticity of the blood vessels. These alterations led to an increase in both Young’s modulus and wall thickness of the renal arteries. In this study, an additional contraction in lumen radius, together with increases in Young’s modulus and wall thickness, was applied in the proximal renal arteries to model HKD [17], with details in Table 2. This additional contraction in the interlobular artery (Fig. 1f) was derived by applying a 10% scaling in PVR relative to DM + HTN baseline.

### Virtual Clinical Trials

This section coupled the vascular model with distinct parameterizations to generate virtual patients for the extraction of waveform-derived biomarkers under DKD and HKD pathological conditions. A logistic regression model was subsequently employed to evaluate the diagnostic utility of these biomarkers through both univariate and multivariate analyses.

#### Generation of Virtual Healthy Controls and Patients

To generate sex- and age-specific virtual healthy controls, CO and all the mechanical parameters of each blood vessel were assumed to be mutually independent. These normalized parameters were randomly and independently assigned in the age-specific model, by sampling each value for each blood vessel from its respective Gaussian distribution. Age-specific male or female subjects were generated by multiplying the dimensional parameters of the 20–29 yo male or female model by the normalized values for CO and mechanical properties across all blood vessels in the age-specific model.

The generated virtual healthy controls comprised 24,000 individuals, categorized by sex (male and female) and six distinct age groups ranging from 20 to 79 yo, with each subgroup containing 2000 individuals. To maintain physiological validity, a filtering process was implemented to exclude any subjects whose mean systolic (SBP) or diastolic (DBP) brachial blood pressure values deviated from the 99% confidence interval (more than 2.575 standard deviations) of the experimentally determined mean [47], and threshold of RI [48], in various sex- and age-specific virtual controls. These physiological filters included 6188 physiological cases from the 24,000 generated virtual healthy controls, resulting in an average inclusion rate of 26%.

To generate virtual patients representing patients with DM + HTN, parameters of each healthy subject from 12 sex- and age-specific virtual controls were scaled by multiplying them by the normalized values for CO and mechanical properties across all blood vessels (Table 2). This resulted in an equal number of virtual patients per sex- and age-specific group as in the corresponding virtual healthy controls. Utilizing the same methodological framework, each virtual patient with DM + HTN was scaled by multiplying their mechanical parameters by the normalized values across corresponding blood vessels in the DKD and HKD model, to generate the sex- and age-specific DM + HTN + DKD and DM + HTN + HKD patients.

The sex- and age-specific virtual healthy controls were validated against *in vivo* literature data for SBP and DBP in the brachial artery, the mean RI across 10 segmental renal arteries, and the mean renal blood flow (RBF) rate in the main renal artery. The DM + HTN [49], DM + HTN + DKD, and DM + HTN + HKD virtual patients were validated against *in vivo* literature data for the mean RI across 10 segmental renal arteries.

#### Biomarkers Extraction

This study translates clinically accessible renal hemodynamic waveforms into quantitative biomarkers. Currently, RI and PI are quantified with Doppler US [49–51], whereas renal blood velocity and volumetric flow rate are measured with PC-MRI [52]. PWV can also be derived with 4D PC-MRI, although its application to the renal artery is not well established [53]. Finally, renal–artery pressure is not reliably obtainable by MRI in clinical practice and is consequently measured invasively with pressure wires [54, 55].

The candidate biomarkers in this study were computed from the full cardiac cycle waveforms of RBF rate, velocity, pressure, and PWV in the main, segmental, interlobar, and arcuate renal arteries for each virtual patient. From each waveform, peak systolic, end diastolic, and cycle mean value defined as the arithmetic mean across a cardiac cycle were computed as candidate biomarkers. RI and pulsatility index (PI) were computed for the left and right renal networks according to Eqs. 1 and 2. Furthermore, systolic acceleration and diastolic deceleration slope of RBF rate, velocity, pressure, and PWV waveform were computed to serve as additional candidate biomarkers, as detailed in Eqs. 3 and 4.

For ease of future reference, biomarkers extracted during the peak systolic phase, end diastolic phase, and mean value were denoted by “Phase name” followed by “Parameter name.” Biomarkers extracted as systolic acceleration slope and diastolic deceleration slope were denoted by “Acceleration” and “Deceleration,” followed by “Parameter name.”

1 $$RI=\frac{{V}_{PSV}-{V}_{EDV}}{{V}_{PSV}},$$

where ${V}_{PSV}$ is the peak systolic blood velocity, and ${V}_{EDV}$ is the end diastolic blood velocity.

2 $$PI=\frac{{V}_{PSV}-{V}_{EDV}}{{V}_{Mean}},$$

where ${V}_{Mean}$ is the mean blood velocity.

3 $$S\text{ystolic acceleration slope }= \frac{{Y}_{PS}-{Y}_{b}}{{t}_{PS}-{t}_{b}},$$

where ${Y}_{PS}$ is the waveform value at the peak systolic phase of the cardiac cycle, ${Y}_{I}$ is the waveform value at the beginning of the systolic upstroke, ${t}_{PS}$ is the time at the peak systolic phase of the cardiac cycle, and ${t}_{b}$ is the time at the beginning of the systolic upstroke.

4 $$Diastolic\;deceleration\;slope\; = \;\frac{{Y_{PS} - Y_{ED} }}{{t_{PS} - t_{ED} }},$$

where ${Y}_{PS}$ is the waveform value at the peak systolic phase of the cardiac cycle, ${Y}_{ED}$ is the waveform value at the end diastolic phase of the cardiac cycle, ${t}_{PS}$ is the time at the peak systolic phase of the cardiac cycle, and ${t}_{ED}$ is the time at the end diastolic phase of the cardiac cycle.

#### Univariate and Multivariate Logistic Regression Model

Logistic regression was chosen as the primary model due to its superior performance over support vector machine, random forest, and decision tree in preliminary analyses of virtual patients with DKD and HKD. Firstly, a univariate analysis was conducted to evaluate their diagnostic potential and to identify optimal measurement locations with strong performance across different sex and age groups. This process involved training and validating a logistic regression model on one biomarker at a time, based on biomarkers extracted from virtual patients. This model used 70% of the data for training and 30% for validation, and the procedure was repeated over 50 random sampling iterations. Model performance was assessed using the AUC, as determined by ROC curve analysis, along with accuracy.

Secondly, a correlation analysis was performed on the biomarkers extracted from the optimal measurement location in the univariate analysis. This analysis assessed the degree of association between biomarkers to understand potential redundancy and multicollinearity in the subsequent multivariate analysis.

Thirdly, a multivariate analysis was performed to identify the high-performing biomarker combinations at the optimal measurement location. The biomarkers were divided into two groups: the first included all available biomarkers and was referred to as the *Full Biomarkers Group*; the second included commonly used biomarkers, excluding pressure- and PWV-related biomarkers and was referred to as the *Common Biomarker Group*. The dimensionality of the biomarker combinations was progressively increased from 2 to 25. For each level of dimensionality, all possible biomarker combinations were randomly generated and evaluated using the same training and cross-validation procedure as applied in the univariate analysis. The biomarker weights, also referred to as logistic regression coefficients, were calculated for each biomarker within each combination. The 10 biomarkers most frequently appearing among the top 100 highest-performing combinations with an AUC greater than 0.8 were identified and counted at each dimensionality level.

## Results

### Validation of Sex- and Age-Specific Virtual Controls

**Fig. 3:**
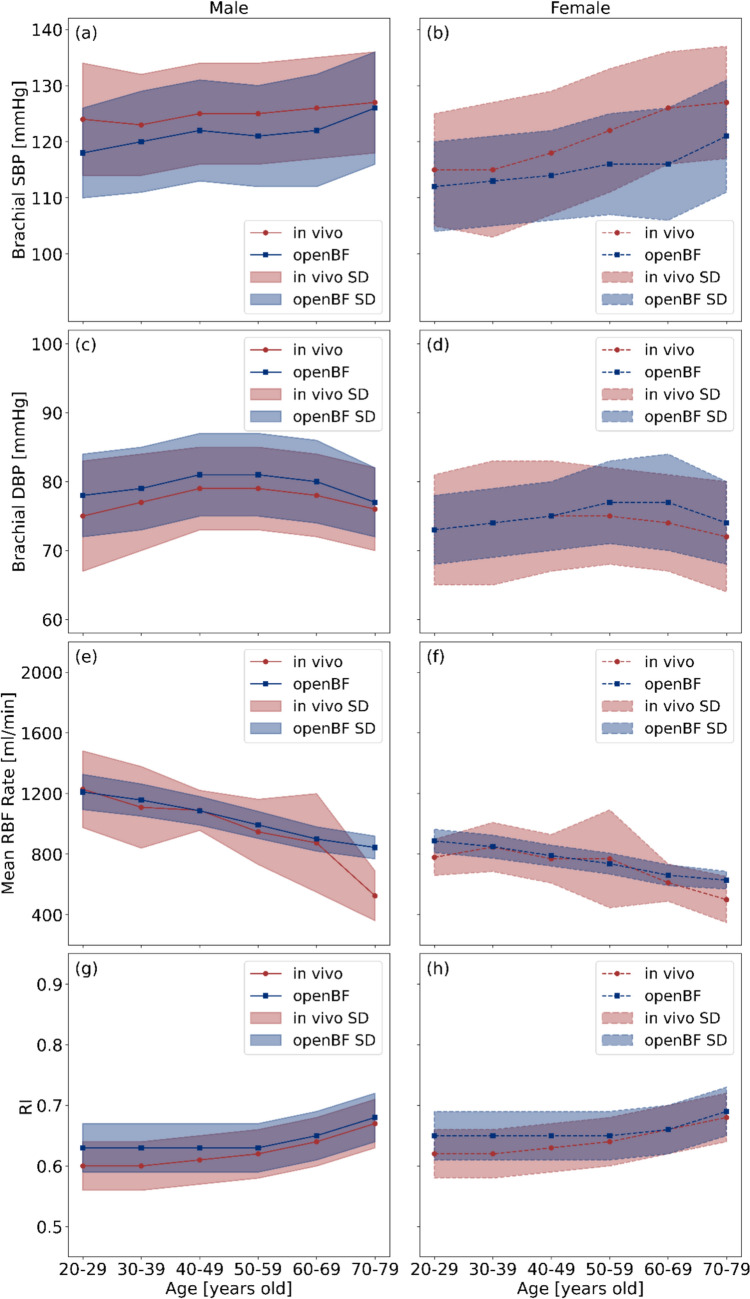
Validation of openBF predictive results in sex- and age-specific virtual healthy controls against *in vivo* literature data, **a**–**d**: comparison of modeled SBP and DBP in the brachial artery against *in vivo* data [47], **e**–**f**: comparison of modeled mean RBF rate in main renal arteries against *in vivo* data [56], **g**–**h**: comparison of RI distributions in the segmental renal artery against *in vivo* data [48]

Fig. 3 presents the validation of openBF predictive results against *in vivo* literature data across various sex- and age-specific virtual healthy controls, with all predictions lying within an acceptable range of variation. In males, SBP ranges from approximately 118 mmHg in the 20–29 age group to around 126 mmHg in the 70–79 group. A similar trend is observed in females, with SBP rising from about 112 mmHg to 121 mmHg across the same age group. In contrast, DBP remains relatively constant with age, averaging around 78 mmHg in males and 75 mmHg in females. Furthermore, male subjects exhibit a decrease in the mean RBF rate from around 1210 ml/min in the 20–29 age group to approximately 844 ml/min in the 70–79 age group. In comparison, female subjects show a similar downward trend, with the mean RBF rate declining from about 887 ml/min to 576 ml/min. In addition, in males, RI increases from around 0.61 in the 20–29 age group to about 0.65 in the 70–79 age group, while in females, RI increases from approximately 0.63 to 0.69.

### Validation of Virtual Patients

**Fig. 4:**
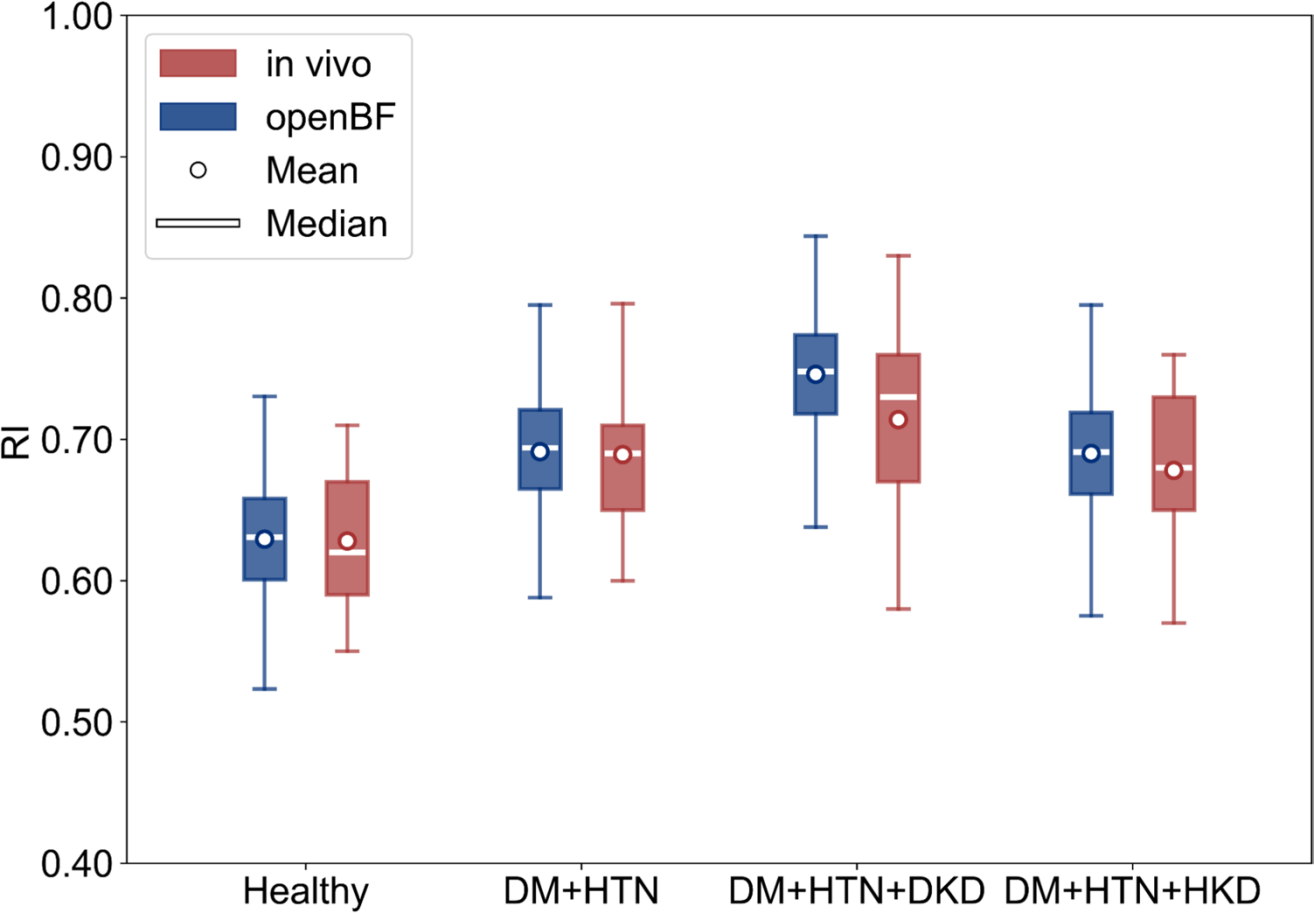
Validation of RI in segmental renal arteries against *in vivo* literature data across populations of healthy [48], DM + HTN [49], DM + HTN + DKD, and DM + HTN + HKD [51], the percentage difference is determined as the absolute difference between the openBF prediction and the *in vivo* data, divided by their mean and expressed as a percentage

Figure 4 presents the comparison of RI between openBF predictive results and *in vivo* literature data for healthy, DM + HTN, DKD (with DM + HTN), and HKD (with DM + HTN) populations. The largest discrepancy between the openBF results and *in vivo* data is observed in the upper whisker of the DKD group, where the openBF result is 0.72 compared to 0.67 from *in vivo* data, resulting in a percentage difference of 7.19%. Furthermore, virtual patients with DM + HTN, even in the absence of explicitly parameterized kidney disease, exhibit an elevated RI, with a mean value of 0.69 compared to 0.63 in healthy individuals. In addition, among the kidney disease groups, virtual patients with HKD present a mean RI comparable to DM + HTN, while DKD exhibits the highest mean RI at 0.74.

### Univariate Analysis

**Fig. 5:**
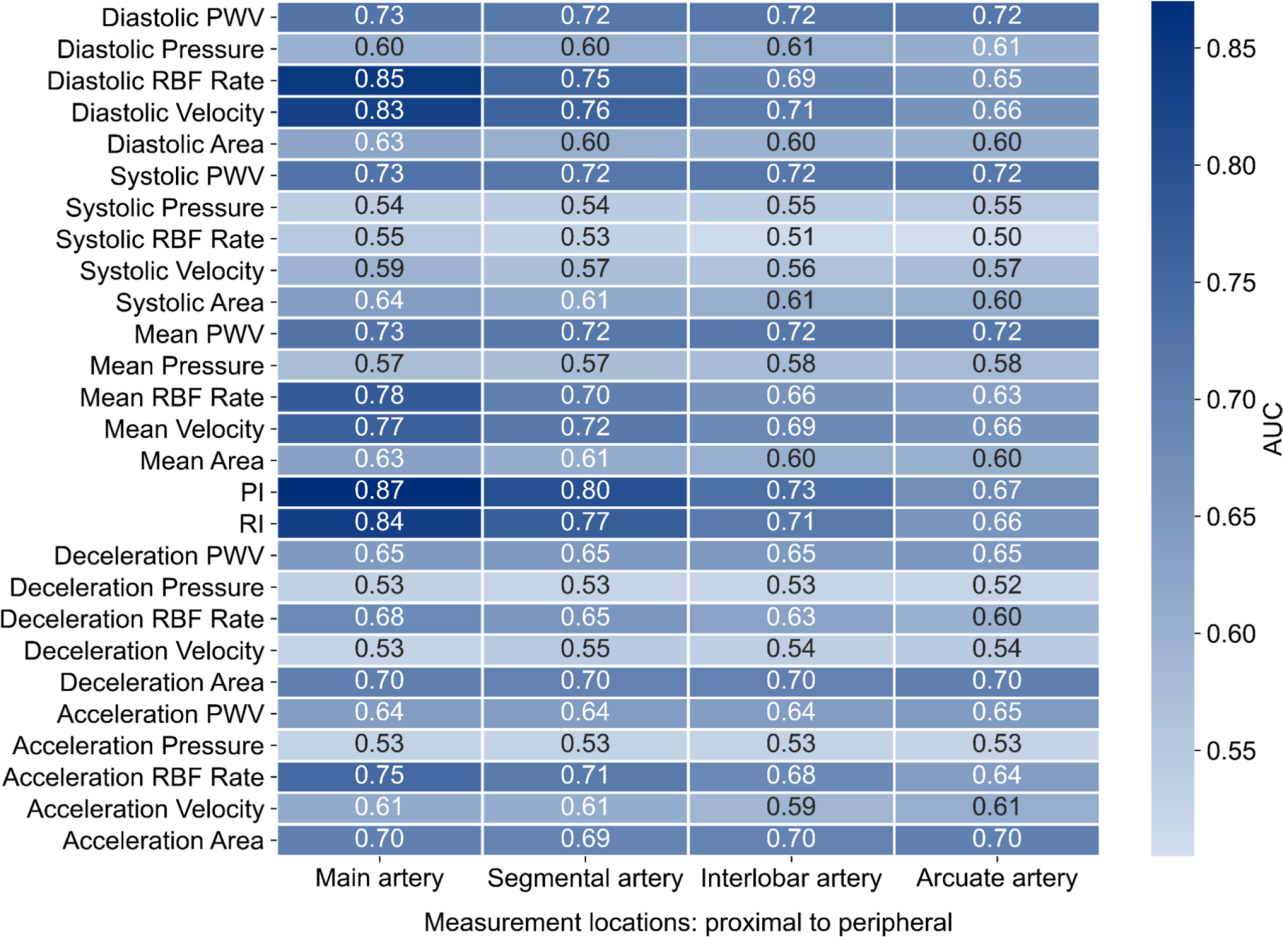
AUC for biomarkers across different renal arteries in a 50–59 yo male virtual patients

Figure 5 presents that velocity- and flow-related biomarkers, particularly PI, achieve the highest AUC values in the main renal artery. However, their diagnostic performance declines progressively from the main renal artery (proximal) to the arcuate artery (peripheral). In contrast, biomarkers associated with PWV, pressure, and area maintain relatively stable diagnostic performance across the renal vascular network. For instance, diastolic PWV, diastolic pressure, and diastolic area exhibit AUC values of approximately 0.73, 0.60, and 0.63, respectively, across the renal vascular network. Conversely, biomarkers derived from the slopes of acceleration and deceleration consistently show low diagnostic performance across the renal vascular network. Similar trends are observed in AUC results for the remaining sex- and age-specific virtual patients, as detailed in the supplementary material.

### Correlation Analysis

**Fig. 6:**
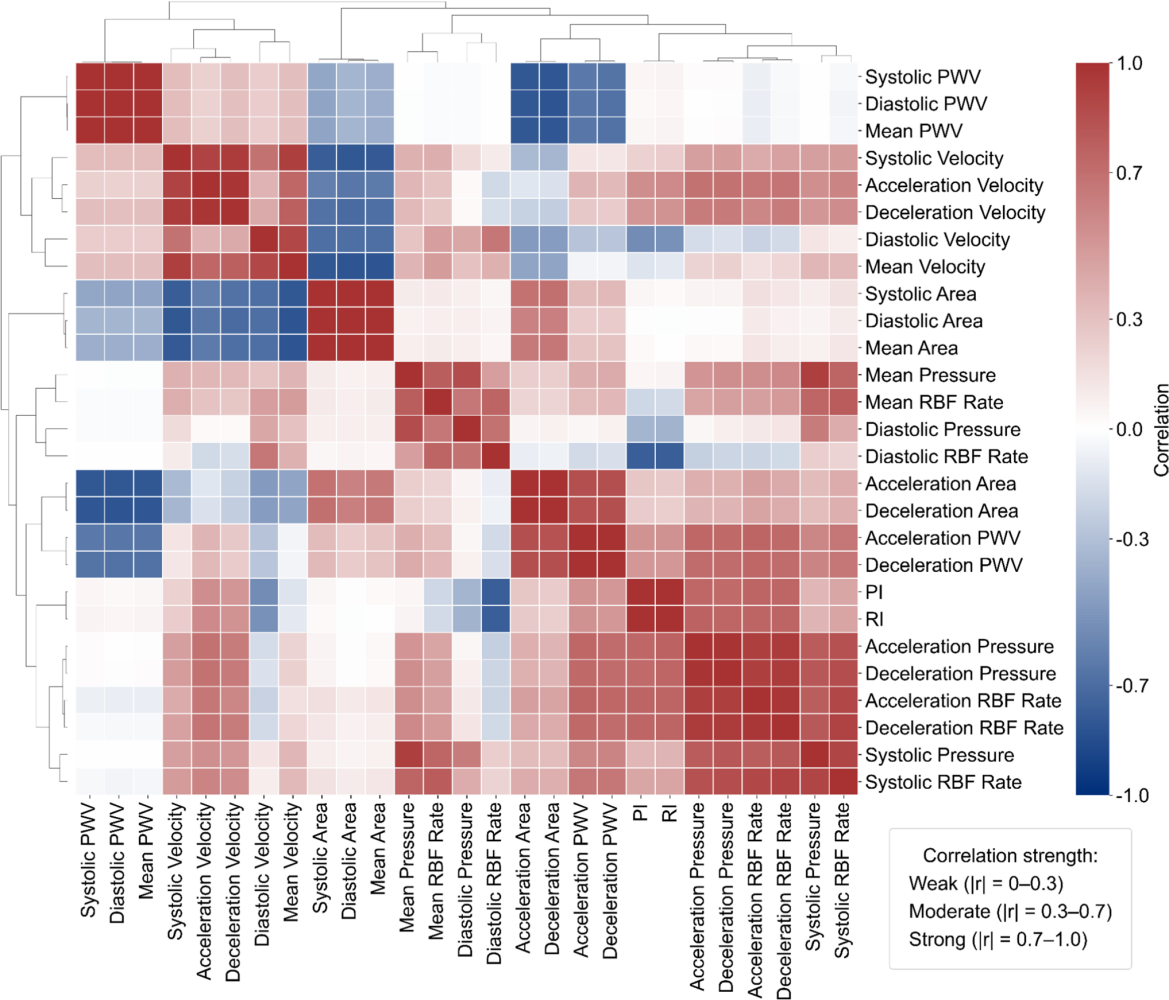
Pearson correlation coefficients between biomarkers measured in the main renal artery for a 50–59 yo male virtual patients

Figure 6 presents clustering patterns among the biomarkers. The phases of RBF Rate (mean, systolic, and diastolic) and the slopes of RBF Rate (acceleration and deceleration) are not directly clustered but belong to separate groups within the same broader cluster. Furthermore, clusters of flow-related biomarkers show moderate to strong correlations with pressure-related biomarkers, and velocity-related biomarkers are moderately clustered with PWV-related biomarkers.

One cluster includes biomarkers such as mean RBF rate and mean pressure, which show strong positive correlations (*r* = 0.78). In contrast, mean velocity and mean area display strong negative correlations (*r* = − 0.81), and RI and diastolic pressure exhibit moderate negative correlations (*r* = − 0.37). Figure 6 also highlights variable pairs with weak correlations, suggesting minimal linear association between the respective pairs, such as RI and Systolic PWV (*r* = 0.06) and diastolic pressure and deceleration velocity (*r* = 0.03).

### Multivariate Analysis

**Fig. 7:**
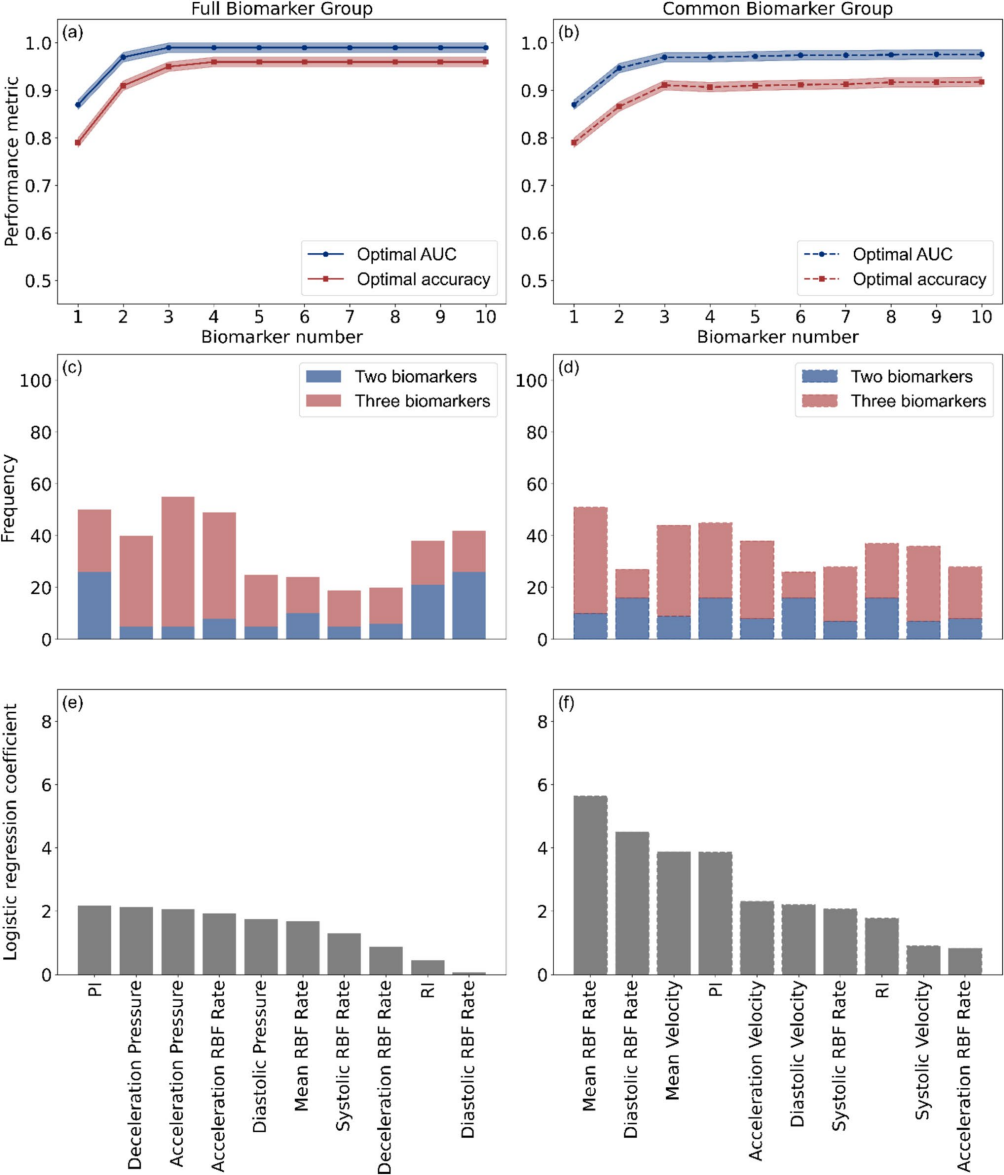
Multivariate analysis of biomarker combinations using the *Full Biomarker Group* and *Common Biomarker Group*, **a** optimal AUC and accuracy as the number of biomarkers increases in the classifier trained on the *Full Biomarker Group*, **b** optimal AUC and accuracy as the number of biomarkers increases in the classifier trained on the *Common Biomarker Group*, **c** top ten most frequently selected biomarkers among top one hundred high-performing two- and three-biomarker combinations in the classifier trained on the *Full Biomarker Group*, **d** top ten most frequently selected biomarkers among top one hundred high-performing two- and three-biomarker combinations in the classifier trained on the *Common Biomarker Group*, **e** logistic regression coefficients of the most frequently selected biomarkers in the classifier trained on the *Full Biomarker Group*, **f** logistic regression coefficients of the most frequently selected biomarkers in the classifier trained on the *Common Biomarker Group*

Figure 7 presents the multivariate analysis of biomarker combinations, highlighting model performance, biomarker selection frequency, and logistic regression weights. When using biomarkers in the *Full Biomarker Group* (Fig. 7a), the model achieves an AUC of 0.97 and an accuracy of 0.91 with two biomarkers. With three biomarkers, the model achieves an improved AUC of 0.99 and an accuracy of 0.95. Adding a fourth biomarker results in only marginal improvement, with both AUC and accuracy converging at 0.93. When using biomarkers in the *Common Biomarker Group* (Fig. 7b**)**, a similar trend is observed, with AUC and accuracy at each dimensionality of the biomarker combination slightly lower than those achieved using the *Full Biomarker Group*.

Figure 7c and d present that acceleration pressure is the most frequently selected biomarker in the *Full Biomarker Group* (55 combinations), and mean RBF rate appears in the most combinations in the *Common Biomarker Group* (51 combinations). In the two-biomarker combinations, PI, diastolic RBF rate, RI, and mean RBF rate exhibit comparable selection frequencies across both the *Full Biomarker Group* and *Common Biomarker Group*.

Figure 7e and f present the biomarker weights derived from logistic regression models for the most frequently selected biomarkers. Mean RBF rate and PI emerge as the most influential biomarkers in both classifiers trained on the *Full Biomarker Group* and the *Common Biomarker Group* and also appear as the most frequently selected biomarkers in Fig. 7c and d. Furthermore, Table 3 shows that classifiers trained using both groups achieve comparable AUC and accuracy, with a maximum AUC difference of 0.02 and an accuracy difference of 0.04.

**Table 3:** Detailed results for top five high-performing two- and three-biomarker combinations in the classifiers trained on the *Full Biomarker Group* and *Common Biomarker Group*

Biomarkers			Metrics	
Biomarker 1	Biomarker 2	Biomarker 3	AUC	Accuracy
*Full Biomarker Group*				
Acceleration RBF Rate	Acceleration Pressure	–	0.97	0.91
Acceleration RBF Rate	Deceleration Pressure	–	0.97	0.91
Acceleration Pressure	PI	–	0.96	0.88
Diastolic Pressure	Diastolic RBF Rate	–	0.95	0.88
Deceleration Pressure	PI	–	0.95	0.87
Systolic Pressure	PI	Diastolic Pressure	0.99	0.95
Deceleration Pressure	PI	Diastolic Pressure	0.99	0.95
Mean Pressure	PI	Deceleration Pressure	0.99	0.95
Acceleration Pressure	PI	Diastolic Pressure	0.99	0.95
Systolic Pressure	PI	Deceleration Pressure	0.99	0.95
*Common Biomarker Group*				
PI	RI	–	0.95	0.87
Mean Velocity	Acceleration Velocity	–	0.92	0.84
Systolic Velocity	Mean Velocity	–	0.92	0.84
Mean RBF Rate	Acceleration RBF Rate	–	0.92	0.83
Systolic RBF Rate	Mean RBF Rate	–	0.91	0.83
Mean RBF Rate	Systolic RBF Rate	Diastolic RBF Rate	0.97	0.91
Mean RBF Rate	Acceleration RBF Rate	Diastolic RBF Rate	0.96	0.89
Mean RBF Rate	Systolic RBF Rate	Deceleration RBF Rate	0.96	0.89
Mean RBF Rate	PI	Diastolic RBF Rate	0.96	0.88
Mean Velocity	Systolic Velocity	Diastolic Velocity	0.95	0.88

### Potential Biomarkers in DKD and HKD Models

**Fig. 8:**
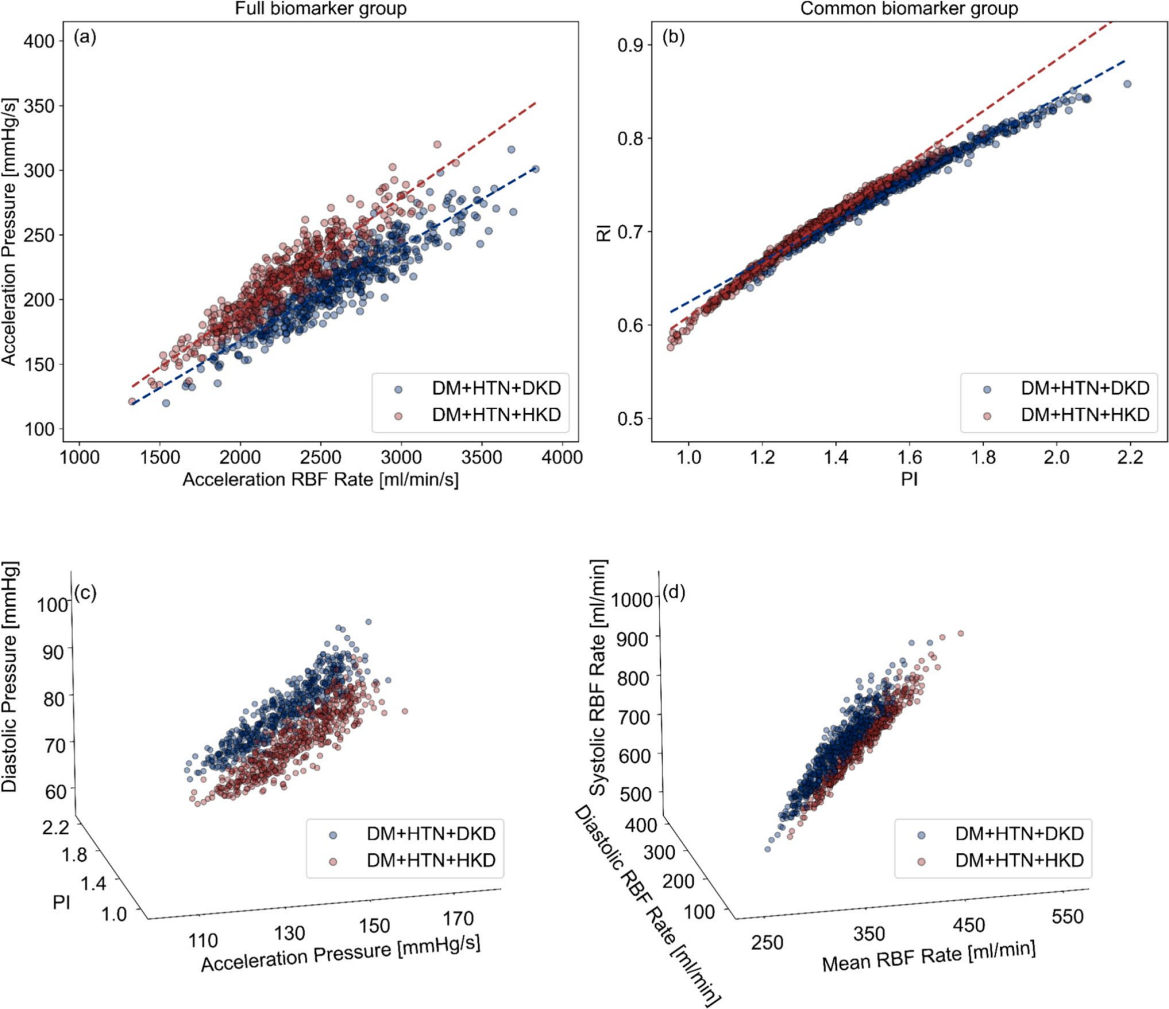
Scatter plot of the optimal diagnostic performance using two- and three-biomarker combinations in a 50–59 yo male virtual patients with DM + HTN + DKD and DM + HTN + HKD; the dark blue point represents DKD patients, while the dark red point represents HKD patients

Fig. 8 presents the optimal performance two- and three-biomarker combinations from the *Full* and *Common Biomarker Group*, respectively. In Fig. 8a, the DKD and HKD groups are relatively well stratified, whereas Fig. 8b shows a slightly tighter clustering of data points around the best-fit curve. Within the Full Biomarker Group, HKD generally exhibits a higher acceleration RBF rate than DKD, while acceleration pressure is similar in both groups. Within the Common Biomarker Group, RI tends to be marginally higher in DKD than in HKD, contributing to the tighter scatter. Furthermore, Fig. 8c shows more pronounced stratification along the PI axis (y-axis), indicating greater variability when PI is included. In contrast, Fig. 8d, the points are more tightly aligned, with stratification primarily driven by the mean RBF rate (x-axis).

## Discussion

This study establishes a multidimensional computational framework for modeling systemic and renal circulation hemodynamics, integrating physiological variations across sex- and age-specific, as well as pathology in renal vasculature associated with DKD and HKD. This study aims to evaluate the hypothesis that divergent systemic and microvascular variants arising from distinct pathophysiological mechanisms of DKD and HKD can be leveraged for an early diagnostic application through cardiovascular biomarkers derived from blood flow waveform analysis.

The results presented in Fig. 3 indicate the predictive accuracy of openBF as validated using virtual healthy controls. The results highlight that trends in brachial SBP and DBP, mean RBF rate, and RI with aging in both male and female are closely consistent with those observed from *in vivo* studies [47, 48, 56]. Our findings reveal marked sex-specific differences in key parameters, including CO, lumen radius, Young’s modulus, and wall thickness, as shown in Fig. 1. The smaller lumen radius in females may contribute to higher PVR, leading to elevated blood pressure [25, 57–59]. However, males generally exhibit higher systemic blood pressure compared to females, as elevated blood pressure is more significantly influenced by higher CO and increased Young’s modulus, which are typically observed in males [60, 61]. These sex-specific differences also affect mean RBF rate and RI values to varying degrees, underscoring the necessity of incorporating sex-specific vascular parameters to accurately capture hemodynamic variability. Although we parameterize the sex factor in our model and validate the sex-specific virtual healthy controls against certain *in vivo* studies, the sex factor remains a critical consideration in clinical research [62] due to physiological and mechanical differences in the cardiovascular system. However, sex factors can be overlooked in some modeling studies [18, 63, 64] when they are not the primary research focus.

The results presented in Fig. 4 indicate that in the absence of diagnosed kidney disease, patients with DM + HTN exhibit elevated RI values compared to healthy individuals, aligning with trends observed from some *in vivo* data [49, 65]. These RI values are not as high as those observed in some patients diagnosed with DKD or HKD [51, 66, 67]. This suggests that alterations in systemic vascular properties can influence renal hemodynamics by affecting blood velocity waveforms, thereby contributing to elevated RI. This is consistent with findings from Madsen’s study, which reported that increased vascular stiffness is associated with impaired diastolic function [68]. Furthermore, RI is approximately 10% higher in virtual patients with DKD than in DM + HTN, while HKD remains comparable to DM + HTN. The increase in RI in DKD patients is due to a greater increase in PVR, caused by contraction of the efferent arteriole and a decrease in nephron number, compared with HKD patients, whereas comparable RI in HKD reflects greater large-artery stiffness and wall thickening with a modest increase in PVR, which raises V_PSV_ and only slightly lowers V_EDV_, keeping RI close to that in DM + HTN. RI (mean = 0.74, SD = 0.04) in the DKD virtual patients exhibits a distribution consistent with a clinical study by Li et al. (RI = 0.70, SD = 0.07), in which all patients are diagnosed with DKD via kidney biopsy and 71% had coexisting systemic DM and HTN [66]. However, both Hashimoto et al. (RI = 0.65, SD = 0.07) and Kawai et al. report lower RI values in HTN patients with renal impairment compared with RI observed in our virtual patients with HKD [51, 67]. The higher RI predicted by openBF may be attributed to only 21% of patients in this study diagnosed with coexisting systemic DM and HTN.

Biomarker performance is highly location-dependent, underscoring the importance of measurement location in clinical applications. As shown in Fig. 5, velocity-related biomarkers, such as PI, diastolic RBF rate, RI, and diastolic velocity, show better diagnostic performance than other types of biomarkers. However, their diagnostic performance decreases progressively from the main renal artery (proximal) to the arcuate artery (peripheral). These results suggest that velocity-related biomarkers are less effective in detecting pathophysiological alterations in peripheral arteries, potentially due to complex hemodynamic conditions or reduced sensitivity in these regions, highlighting the regional dependence of their diagnostic performance. This finding corroborates established clinical practices in which velocity-related biomarkers, such as PI and RI, are typically assessed in the proximal segments of renal vasculature for diagnosing kidney disease [69, 70]. The diminished diagnostic performance in more peripheral regions may be attributed to local hemodynamic influences, including increased microvascular resistance and complex branching patterns, which attenuate the effectiveness of velocity-related biomarkers [71]. These findings underscore the necessity of location-specific biomarker selection to improve diagnostic accuracy and guide more targeted interventions in kidney disease.

The correlation structure groups biomarkers into physiologically coherent clusters relevant to disease discrimination. Specifically, PWV indicates arterial stiffness, while velocity and RBF capture intrarenal perfusion. In addition, the lumen area reflects vessel size and compliance, with a lower area indicating constriction or reduced compliance. Moreover, pressure indices quantify upstream driving pressure and hemodynamic load. Finally, RI and PI characterize downstream microvascular resistance and pulsatility. These clusters imply that multivariate models gain by combining stiffness, perfusion, compliance, driving pressure, and downstream resistance rather than relying on near-duplicate signals. Consistently, top-performing pairs couple pressure with flow or velocity, capturing the driving force and the vascular response and approximating the pressure-flow relationship and vascular impedance to improve discrimination.

The optimal performance of biomarker combinations using the *Full Biomarker Group* is slightly higher than that of combinations derived from only the *Full Biomarker Group*. In both cases, performance shows relatively small improvement beyond three biomarkers and converges when four biomarkers are used. This trend suggests that the inclusion of additional biomarkers may initially enhance AUC and accuracy, possibly by increasing the model’s capacity to represent variation associated with group differences. However, the marginal gain diminishes as the added biomarkers contribute increasingly redundant or non-informative features.

These virtual clinical trials can improve cost-effectiveness in clinical trials by optimizing scan protocols and prioritizing candidate biomarkers, thereby focusing trial resources (scanning time and study budget) on the most informative biomarker panels. Because kidney biopsy is rarely performed to diagnose early-stage disease, these non-invasive imaging biomarkers could be used as adjuncts to refine pretest probability and support risk stratification. They are not positioned to replace biopsy at present. If their discrimination performance is confirmed prospectively, they could support biopsy decisions by identifying equivocal cases and deprioritizing biopsy in clearly low-risk patients, thereby reducing unnecessary invasive procedures. Because flow-based indices capture vascular dysfunction before overt structural change, they are expected to be useful in earlier disease stages and for monitoring treatment response, with stage-specific thresholds defined through prospective validation.

This study also has some limitations that need to be considered. Firstly, the current framework does not incorporate variability beyond age and sex, including ethnicity, medication effects, anthropometric characteristics (body weight, height, body surface area), and interindividual variation in nephron number (approximately 0.6 to 1.2 million per kidney). These determinants shape vascular and nephron-scale structure and function and can influence biomarker performance. Future work should incorporate these covariates using allometric scaling to link them to vascular properties within the model and literature-informed priors [72, 73], to improve personalization and enhance the generalizability of the proposed biomarkers.

Secondly, the renal microcirculation is represented with a lumped R-C-R parameter model implemented as an equivalent circuit. This circuit is not coupled to the proximal renal circulation and does not explicitly include renal autoregulatory mechanisms. This simplification constrains accurate representation of dynamic interactions between the proximal circulation and the renal microcirculation under pathological conditions. Future work should improve physiological fidelity by detailing the proximal renal vasculature and coupling it to microvascular compartments, incorporating renal autoregulation, and modeling kidney disease progression [15, 74].

Thirdly, a total of 6188 physiological cases were retained, representing 26% of the 24,000 generated virtual healthy controls. This low inclusion rate is mainly due to independent random sampling. Although each parameter falls within physiological ranges, selecting them independently can produce unphysiological combinations. Future work could use correlation-constrained sampling. At the arterial tree level, one could apply physiology-consistent constraints, by enforcing Murray’s law for branching and radius scaling [75], standardize the coupling between the lumen and the wall using a single tube law derived from thin wall mechanics [76], and apply a proximal-to-distal elasticity gradient to regularize wall parameters [77]. By reducing degrees of freedom and limiting parameter degeneracy, the proportion of physiologically implausible virtual controls should decrease substantially.

The diagnostic performance of the proposed biomarkers in differentiating DKD from HKD currently lacks *in vivo* validation, which limits clinical translatability. Future work should focus on patient cohorts mirroring the modeled scenarios, specifically patients with coexisting DM and HTN who are clinically classified as DKD or HKD, with CKD stage 2 confirmed by laboratory blood and urine tests. The same biomarkers identified in this study should be acquired using the appropriate imaging modalities. Clinical validation could then apply logistic regression with ROC analysis, replicating the approach described in this study. Translation into clinical practice is constrained by the lack of a gold standard to distinguish DKD from HKD. Although kidney biopsy serves as the reference test, the procedure is invasive, carries bleeding risk, and is rarely undertaken in early CKD, while clinical criteria are often nonspecific with overlapping phenotypes. Furthermore, routine clinical application will require standardized acquisition protocols across scanners and sites, reproducible waveform extraction, and rigorous signal quality control.

In conclusion, this study highlights the potential of hemodynamic-related biomarkers, combined with a multidimensional mechanistic modeling and machine learning-based approach, to distinguish between DKD and HKD. Our findings show that proximal renal arteries, such as the main renal artery and segmental renal artery, are the optimal locations for obtaining these biomarkers. Furthermore, the utilization of two to three biomarkers with moderate correlation can improve the diagnostic accuracy for distinguishing DKD from HKD. Among these biomarkers, Mean RBF Rate and PI serve as the foundational biomarkers, appearing most frequently in high-performing combinations and accessible with routine clinical measurements. This study represents a notable advancement in kidney disease diagnostics, offering a modeling and non-invasive method for addressing two complex and overlapping pathologies.

## Supplementary Information

Below is the link to the electronic supplementary material.

Supplementary file1 (DOCX 1570 KB)
