# Supplementary material for: A Virtual Trial to Identify Cardiovascular Biomarkers for Differentiating Diabetic and Hypertensive Kidney Disease
Source: Ann Biomed Eng. 2026 Jan 29;54(6):1746–64. doi: 10.1007/s10439-026-03983-4 (PMC13186901; doi:10.1007/s10439-026-03983-4)
Supplement: Supplementary file 1 — Supplementary file1 (DOCX 1570 KB) [file 10439_2026_3983_MOESM1_ESM.docx]

**Supplementary materials for:**

**A Virtual Trial to Identify Cardiovascular Biomarkers for Differentiating Diabetic and Hypertensive Kidney Disease**

Ning Wang^1,2^, Steven P Sourbron^1,3^, Ivan Benemerito^1,2^, Alberto Marzo^1,2^

^1^ INSIGNEO Institute for *in silico* medicine, University of Sheffield, Sheffield, UK

^2^ School of Mechanical, Aerospace and Civil Engineering, University of Sheffield, Sheffield, UK

^3^ School of Medicine and Population Health, University of Sheffield, Sheffield, UK

Correspondence to:

Ning Wang

INSIGNEO Institute for *in silico* medicine & School of Mechanical, Aerospace and Civil Engineering, University of Sheffield, Sheffield, UK

Room E09, The Pam Liversidge Building, Mappin Street, S1 3JD, Sheffield, UK

ning.wang1@sheffield.ac.uk

+44 114 222 6173

# Whole-body circulation baseline model

The 1D whole-circulation network for a healthy subject is illustrated in Fig. S1, with the vessel lengths, lumen radii, Young’s moduli, wall thicknesses, and Windkessel parameters detailed in Table S1. Blood density and viscosity were set to 1,060 kg/m^3^ and 4.00×10^-3^ Pa · s, respectively, to simulate a healthy subject in openBF.


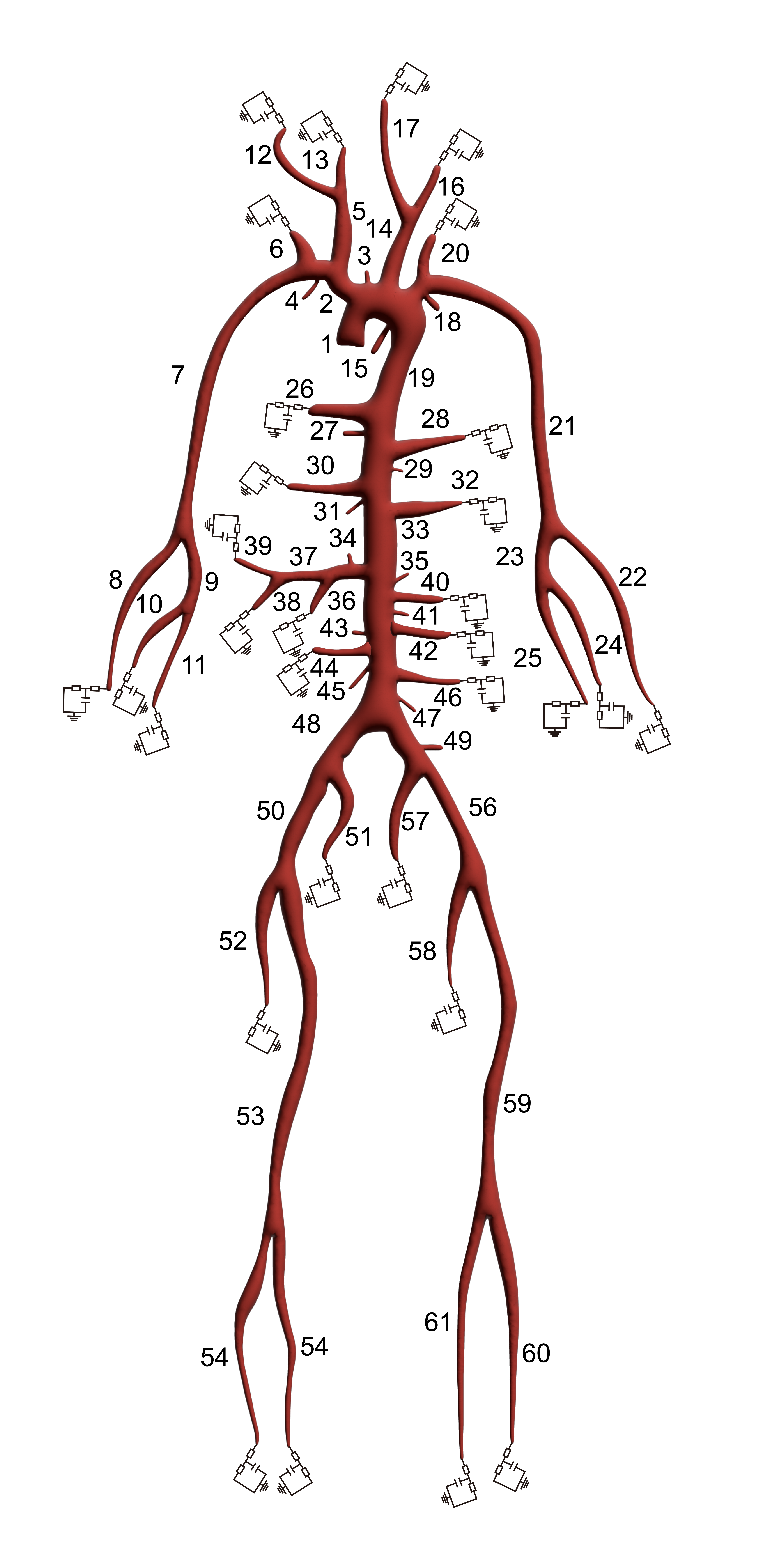


Fig. S1 Illustration of 1D whole-body circulation model

Table S1 Mechanical properties of blood vessels and 0D lumped-parameter R-C-R models used in the 1D whole-body circulation network

| Number | Blood vessel | L  [cm] | R  [mm] | E  [kPa] | h0  [mm] | R1+R2  [Pa·s·m^-3^] | Cc  [m^3^·Pa^-1^] |
| --- | --- | --- | --- | --- | --- | --- | --- |
| 1 | Aortic_arch_I | 7.44 | 15.95 | 350 | 1.77 |  |  |
| 2 | Brachiocephalic_trunk | 4.74 | 6.73 | 350 | 0.89 |  |  |
| 3 | Aortic_arch_II | 0.96 | 12.95 | 350 | 1.49 |  |  |
| 4 | Subclavian_R_I | 1.57 | 4.90 | 350 | 0.73 |  |  |
| 5 | Common_carotid_R | 8.12 | 4.48 | 350 | 0.69 |  |  |
| 6 | Vertebral_R | 20.45 | 1.34 | 350 | 0.37 | 9.77E+09 | 2.88E-11 |
| 7a | Subclavian_R_II | 4.11 | 4.18 | 350 | 0.67 |  |  |
| 7b | Axillary_R | 12.00 | 2.30 | 350 | 0.50 |  |  |
| 7c | Brachial_R | 22.31 | 2.08 | 450 | 0.47 |  |  |
| 8 | Radial_R | 30.09 | 1.38 | 450 | 0.37 | 6.23E+09 | 4.52E-11 |
| 9 | Ulnar_R_I | 2.98 | 1.41 | 450 | 0.38 |  |  |
| 10a | Common_interosseous_R | 1.63 | 0.96 | 450 | 0.29 |  |  |
| 10b | Ulnar_R_II | 23.06 | 0.68 | 450 | 0.22 | 2.58E+10 | 1.09E-11 |
| 11 | Posterior_interosseous_R | 23.93 | 1.41 | 450 | 0.38 | 6.34E+09 | 4.43E-11 |
| 12 | External_carotid_R | 6.09 | 2.27 | 350 | 0.50 | 5.08E+09 | 5.55E-11 |
| 13 | Internal_carotid_R | 13.21 | 2.77 | 350 | 0.55 | 3.11E+09 | 9.04E-11 |
| 14 | Common_carotid_L | 12.13 | 4.48 | 350 | 0.69 |  |  |
| 15 | Aortic_arch_III | 0.70 | 12.57 | 350 | 1.45 |  |  |
| 16 | External_carotid_L | 6.09 | 2.27 | 350 | 0.50 | 5.09E+09 | 5.53E-11 |
| 17 | Internal_carotid_L | 13.21 | 2.77 | 350 | 0.55 | 3.12E+09 | 9.02E-11 |
| 18 | Subclavian_L_I | 4.94 | 4.90 | 350 | 0.73 |  |  |
| 19a | Aortic_arch_IV | 4.31 | 12.28 | 350 | 1.42 |  |  |
| 19b | Thoracic aorta I | 0.99 | 10.55 | 350 | 1.26 |  |  |
| 20 | Vertebral_L | 20.42 | 1.34 | 350 | 0.37 | 1.04E+10 | 2.70E-11 |
| 21a | Subclavian_L_II | 4.11 | 3.48 | 350 | 0.61 |  |  |
| 21b | Axillary_L | 12.00 | 2.30 | 350 | 0.50 |  |  |
| 21c | Brachial_L | 22.31 | 2.08 | 450 | 0.47 |  |  |
| 22 | Radial_L | 31.09 | 1.38 | 450 | 0.37 | 6.12E+09 | 4.60E-11 |
| 23 | Ulnar L I | 2.98 | 1.41 | 450 | 0.38 |  |  |
| 24a | Common interosseous L | 1.63 | 0.96 | 450 | 0.29 |  |  |
| 24b | Posterior interosseous L | 23.06 | 0.68 | 350 | 0.22 | 2.59E+10 | 1.09E-11 |
| 25 | Ulnar L II | 23.93 | 1.41 | 450 | 0.38 | 6.47E+09 | 4.35E-11 |
| 26 | Posterior intercostal R 1 | 19.69 | 1.40 | 450 | 0.38 | 1.35E+11 | 2.09E-11 |
| 27 | Thoracic aorta II | 0.79 | 10.36 | 350 | 1.24 |  |  |
| 28 | Posterior intercostal L 1 | 17.80 | 1.40 | 450 | 0.38 | 1.38E+11 | 2.04E-12 |
| 29 | Thoracic aorta III | 1.56 | 10.22 | 350 | 1.22 |  |  |
| 30 | Posterior intercostal R 2 | 20.16 | 1.55 | 350 | 0.40 | 1.25E+11 | 2.24E-12 |
| 31 | Thoracic aorta IV | 0.53 | 9.92 | 350 | 1.19 |  |  |
| 32 | Posterior intercostal L 2 | 18.52 | 1.55 | 450 | 0.40 | 1.26E+11 | 2.23E-12 |
| 33a | Thoracic aorta V | 12.16 | 9.82 | 350 | 1.18 |  |  |
| 33b | Thoracic aorta VI | 0.33 | 7.54 | 350 | 0.96 |  |  |
| 34 | Celiac trunk | 1.68 | 3.35 | 350 | 0.60 |  |  |
| 35 | Abdominal aorta I | 1.40 | 7.48 | 350 | 0.96 |  |  |
| 36 | Common hepatic | 6.66 | 2.69 | 350 | 0.54 | 1.80E+09 | 1.55E-10 |
| 37 | Common hepatic | 0.40 | 2.17 | 350 | 0.48 |  |  |
| 38 | Left gastric | 9.29 | 1.51 | 350 | 0.39 | 1.86E+11 | 1.52E-12 |
| 39 | Splenic II | 6.44 | 2.17 | 350 | 0.48 | 2.56E+09 | 1.10E-10 |
| 40 | Superior mesenteric | 21.64 | 3.93 | 350 | 0.65 | 1.18E+09 | 2.39E-10 |
| 41 | Abdominal aorta II | 0.43 | 7.32 | 350 | 0.94 |  |  |
| 43 | Abdominal aorta III | 1.20 | 7.26 | 350 | 0.94 |  |  |
| 45 | Abdominal aorta IV | 5.41 | 7.11 | 350 | 0.92 |  |  |
| 46 | Inferior mesenteric | 9.02 | 2.08 | 350 | 0.47 | 1.30E+10 | 2.18E-11 |
| 47 | Abdominal aorta V | 4.22 | 6.43 | 350 | 0.86 |  |  |
| 48 | Common iliac R | 7.64 | 4.50 | 350 | 0.70 |  |  |
| 49 | Common iliac L | 7.40 | 4.50 | 350 | 0.70 |  |  |
| 50a | External iliac R | 10.22 | 3.38 | 350 | 0.60 |  |  |
| 50b | Femoral R I | 3.16 | 3.19 | 450 | 0.59 |  |  |
| 51 | Internal iliac R | 7.25 | 2.82 | 300 | 0.55 | 2.24E+09 | 1.26E-10 |
| 52 | Profunda femoris R | 23.84 | 2.14 | 450 | 0.48 | 1.85E+09 | 1.52E-10 |
| 53a | Femoral R II | 31.93 | 3.14 | 450 | 0.58 |  |  |
| 53b | Popliteal R I | 13.20 | 2.69 | 450 | 0.54 |  |  |
| 54 | Anterior tibial R | 38.62 | 1.17 | 450 | 0.33 | 1.33E+10 | 2.13E-11 |
| 55a | Popliteal R II | 0.88 | 2.37 | 450 | 0.51 |  |  |
| 55b | Tibio bular trunk R | 3.62 | 2.35 | 450 | 0.50 |  |  |
| 55c | Posterior tibial R | 38.29 | 1.23 | 450 | 0.35 | 1.14E+10 | 2.47E-11 |
| 56a | External iliac L | 10.22 | 3.38 | 350 | 0.60 |  |  |
| 56b | Femoral L I | 3.16 | 3.19 | 350 | 0.59 |  |  |
| 57 | Internal iliac L | 7.25 | 2.82 | 350 | 0.55 | 2.25E+09 | 1.25E-10 |
| 58 | Profunda femoris L | 23.84 | 2.14 | 350 | 0.48 | 1.85E+09 | 1.52E-10 |
| 59a | Femoral L II | 31.93 | 3.14 | 450 | 0.58 |  |  |
| 59b | Popliteal L I | 13.20 | 2.69 | 350 | 0.54 |  |  |
| 60 | Anterior tibial L | 38.62 | 1.17 | 350 | 0.33 | 1.33E+10 | 2.13E-11 |
| 61a | Popliteal L II | 0.88 | 2.37 | 450 | 0.51 |  |  |
| 61b | Tibiobular trunk L | 3.62 | 2.35 | 350 | 0.50 |  |  |
| 61c | Posterior tibial L | 38.29 | 1.23 | 450 | 0.35 | 9.15E+09 | 2.47E-11 |

# Renal circulation baseline model

The following renal circulation network illustrated in Fig. S2 for a healthy subject was expanded by connecting it to the above-mentioned 1D whole circulation network (Fig. S1), which extends from the main renal arteries left (42) and right (44) to the arcuate renal arteries. Details including length, lumen radii, vascular Young’s moduli, wall thickness, and Windkessel parameters are provided in Table S2.


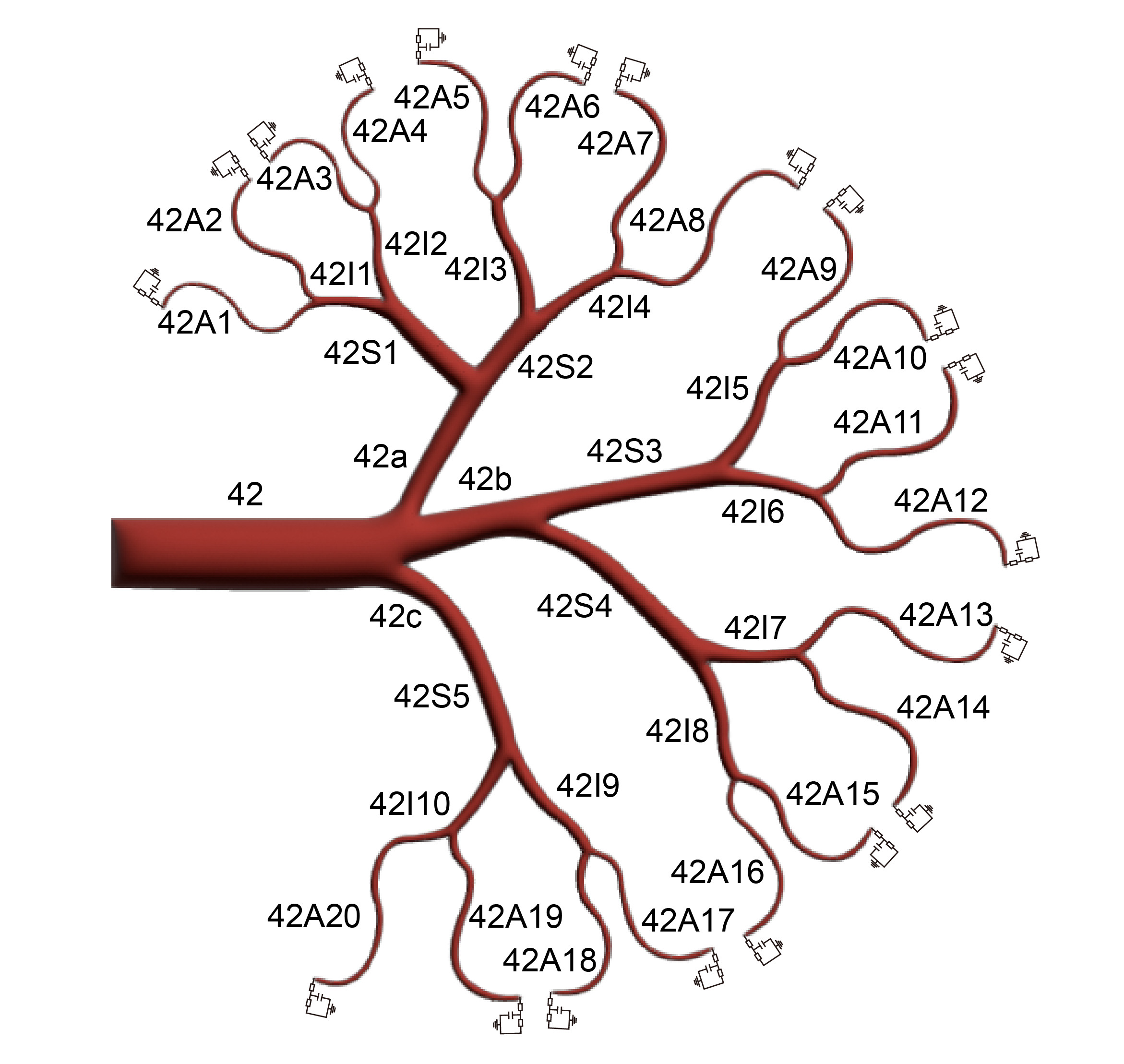


Fig. S2 Illustration of 1D renal circulation

Table S2 Mechanical properties of 1D renal arteries coupled with 0D lumped parameter R-C-R models representing the renal microcirculation

| Number | Blood vessel | L  [cm] | R  [mm] | E  [kPa] | h0  [mm] | R1+R2  [Pa·s·m^-3^] | Cc  [m^3^·Pa^-1^] |
| --- | --- | --- | --- | --- | --- | --- | --- |
| 42 | Renal_L | 2.18 | 3.02 | 450 | 0.57 |  |  |
| 42a | Renal_tri_L | 0.10 | 3.02 | 450 | 0.57 |  |  |
| 42b | Renal_tri_L | 0.10 | 3.02 | 450 | 0.57 |  |  |
| 42c | Renal_tri_L | 0.10 | 3.02 | 450 | 0.57 |  |  |
| 42S1 | Renal_segment_L | 1.40 | 2.39 | 900 | 0.51 |  |  |
| 42S2 | Renal_segment_L | 1.40 | 2.39 | 900 | 0.51 |  |  |
| 42S3 | Renal_segment_L | 1.40 | 2.39 | 900 | 0.51 |  |  |
| 42S4 | Renal_segment_L | 1.40 | 2.39 | 900 | 0.51 |  |  |
| 42S5 | Renal_segment_L | 1.40 | 2.39 | 900 | 0.51 |  |  |
| 42I1 | Renal_interlobar_L | 1.70 | 1.84 | 1350 | 0.44 |  |  |
| 42I2 | Renal_interlobar_L | 1.70 | 1.84 | 1350 | 0.44 |  |  |
| 42I3 | Renal_interlobar_L | 1.70 | 1.84 | 1350 | 0.44 |  |  |
| 42I4 | Renal_interlobar_L | 1.70 | 1.84 | 1350 | 0.44 |  |  |
| 42I5 | Renal_interlobar_L | 1.70 | 1.84 | 1350 | 0.44 |  |  |
| 42I6 | Renal_interlobar_L | 1.70 | 1.84 | 1350 | 0.44 |  |  |
| 42I7 | Renal_interlobar_L | 1.70 | 1.84 | 1350 | 0.44 |  |  |
| 42I8 | Renal_interlobar_L | 1.70 | 1.84 | 1350 | 0.44 |  |  |
| 42I9 | Renal_interlobar_L | 1.70 | 1.84 | 1350 | 0.44 |  |  |
| 42I10 | Renal_interlobar_L | 1.70 | 1.84 | 1350 | 0.44 |  |  |
| 42Ar1 | Renal_arcuate_L | 1.00 | 0.87 | 1800 | 0.27 | 3.22E+10 | 2.53E-11 |
| 42Ar2 | Renal_arcuate_L | 1.00 | 0.87 | 1800 | 0.27 | 3.22E+10 | 2.53E-11 |
| 42Ar3 | Renal_arcuate_L | 1.00 | 0.87 | 1800 | 0.27 | 3.22E+10 | 2.53E-11 |
| 42Ar4 | Renal_arcuate_L | 1.00 | 0.87 | 1800 | 0.27 | 3.22E+10 | 2.53E-11 |
| 42Ar5 | Renal_arcuate_L | 1.00 | 0.87 | 1800 | 0.27 | 3.22E+10 | 2.53E-11 |
| 42Ar6 | Renal_arcuate_L | 1.00 | 0.87 | 1800 | 0.27 | 3.22E+10 | 2.53E-11 |
| 42Ar7 | Renal_arcuate_L | 1.00 | 0.87 | 1800 | 0.27 | 3.22E+10 | 2.53E-11 |
| 42Ar8 | Renal_arcuate_L | 1.00 | 0.87 | 1800 | 0.27 | 3.22E+10 | 2.53E-11 |
| 42Ar9 | Renal_arcuate_L | 1.00 | 0.87 | 1800 | 0.27 | 3.22E+10 | 2.53E-11 |
| 42Ar10 | Renal_arcuate_L | 1.00 | 0.87 | 1800 | 0.27 | 3.22E+10 | 2.53E-11 |
| 42Ar11 | Renal_arcuate_L | 1.00 | 0.87 | 1800 | 0.27 | 3.22E+10 | 2.53E-11 |
| 42Ar12 | Renal_arcuate_L | 1.00 | 0.87 | 1800 | 0.27 | 3.22E+10 | 2.53E-11 |
| 42Ar13 | Renal_arcuate_L | 1.00 | 0.87 | 1800 | 0.27 | 3.22E+10 | 2.53E-11 |
| 42Ar14 | Renal_arcuate_L | 1.00 | 0.87 | 1800 | 0.27 | 3.22E+10 | 2.53E-11 |
| 42Ar15 | Renal_arcuate_L | 1.00 | 0.87 | 1800 | 0.27 | 3.22E+10 | 2.53E-11 |
| 42Ar16 | Renal_arcuate_L | 1.00 | 0.87 | 1800 | 0.27 | 3.22E+10 | 2.53E-11 |
| 42Ar17 | Renal_arcuate_L | 1.00 | 0.87 | 1800 | 0.27 | 3.22E+10 | 2.53E-11 |
| 42Ar18 | Renal_arcuate_L | 1.00 | 0.87 | 1800 | 0.27 | 3.22E+10 | 2.53E-11 |
| 42Ar19 | Renal_arcuate_L | 1.00 | 0.87 | 1800 | 0.27 | 3.22E+10 | 2.53E-11 |
| 42Ar20 | Renal_arcuate_L | 1.00 | 0.87 | 1800 | 0.27 | 3.22E+10 | 2.53E-11 |
| 44 | Renal_R | 3.18 | 2.96 | 450 | 0.56 |  |  |
| 44a | Renal_tri_R | 0.10 | 2.96 | 450 | 0.56 |  |  |
| 44b | Renal_tri_R | 0.10 | 2.96 | 450 | 0.56 |  |  |
| 44c | Renal_tri_R | 0.10 | 2.96 | 450 | 0.56 |  |  |
| 44S1 | Renal_segment_R | 1.50 | 2.35 | 900 | 0.50 |  |  |
| 44S2 | Renal_segment_R | 1.50 | 2.35 | 900 | 0.50 |  |  |
| 44S3 | Renal_segment_R | 1.50 | 2.35 | 900 | 0.50 |  |  |
| 44S4 | Renal_segment_R | 1.50 | 2.35 | 900 | 0.50 |  |  |
| 44S5 | Renal_segment_R | 1.50 | 2.35 | 900 | 0.50 |  |  |
| 44I1 | Renal_interlobar_R | 1.80 | 1.80 | 1350 | 0.44 |  |  |
| 44I2 | Renal_interlobar_R | 1.80 | 1.80 | 1350 | 0.44 |  |  |
| 44I3 | Renal_interlobar_R | 1.80 | 1.80 | 1350 | 0.44 |  |  |
| 44I4 | Renal_interlobar_R | 1.80 | 1.80 | 1350 | 0.44 |  |  |
| 44I5 | Renal_interlobar_R | 1.80 | 1.80 | 1350 | 0.44 |  |  |
| 44I6 | Renal_interlobar_R | 1.80 | 1.80 | 1350 | 0.44 |  |  |
| 44I7 | Renal_interlobar_R | 1.80 | 1.80 | 1350 | 0.44 |  |  |
| 44I8 | Renal_interlobar_R | 1.80 | 1.80 | 1350 | 0.44 |  |  |
| 44I9 | Renal_interlobar_R | 1.80 | 1.80 | 1350 | 0.44 |  |  |
| 44I10 | Renal_interlobar_R | 1.80 | 1.80 | 1350 | 0.44 |  |  |
| 44Ar1 | Renal_arcuate_R | 1.00 | 0.85 | 1800 | 0.27 | 3.22E+10 | 2.53E-11 |
| 44Ar2 | Renal_arcuate_R | 1.00 | 0.85 | 1800 | 0.27 | 3.22E+10 | 2.53E-11 |
| 44Ar3 | Renal_arcuate_R | 1.00 | 0.85 | 1800 | 0.27 | 3.22E+10 | 2.53E-11 |
| 44Ar4 | Renal_arcuate_R | 1.00 | 0.85 | 1800 | 0.27 | 3.22E+10 | 2.53E-11 |
| 44Ar5 | Renal_arcuate_R | 1.00 | 0.85 | 1800 | 0.27 | 3.22E+10 | 2.53E-11 |
| 44Ar6 | Renal_arcuate_R | 1.00 | 0.85 | 1800 | 0.27 | 3.22E+10 | 2.53E-11 |
| 44Ar7 | Renal_arcuate_R | 1.00 | 0.85 | 1800 | 0.27 | 3.22E+10 | 2.53E-11 |
| 44Ar8 | Renal_arcuate_R | 1.00 | 0.85 | 1800 | 0.27 | 3.22E+10 | 2.53E-11 |
| 44Ar9 | Renal_arcuate_R | 1.00 | 0.85 | 1800 | 0.27 | 3.22E+10 | 2.53E-11 |
| 44Ar10 | Renal_arcuate_R | 1.00 | 0.85 | 1800 | 0.27 | 3.22E+10 | 2.53E-11 |
| 44Ar11 | Renal_arcuate_R | 1.00 | 0.85 | 1800 | 0.27 | 3.22E+10 | 2.53E-11 |
| 44Ar12 | Renal_arcuate_R | 1.00 | 0.85 | 1800 | 0.27 | 3.22E+10 | 2.53E-11 |
| 44Ar13 | Renal_arcuate_R | 1.00 | 0.85 | 1800 | 0.27 | 3.22E+10 | 2.53E-11 |
| 44Ar14 | Renal_arcuate_R | 1.00 | 0.85 | 1800 | 0.27 | 3.22E+10 | 2.53E-11 |
| 44Ar15 | Renal_arcuate_R | 1.00 | 0.85 | 1800 | 0.27 | 3.22E+10 | 2.53E-11 |
| 44Ar16 | Renal_arcuate_R | 1.00 | 0.85 | 1800 | 0.27 | 3.22E+10 | 2.53E-11 |
| 44Ar17 | Renal_arcuate_R | 1.00 | 0.85 | 1800 | 0.27 | 3.22E+10 | 2.53E-11 |
| 44Ar18 | Renal_arcuate_R | 1.00 | 0.85 | 1800 | 0.27 | 3.22E+10 | 2.53E-11 |
| 44Ar19 | Renal_arcuate_R | 1.00 | 0.85 | 1800 | 0.27 | 3.22E+10 | 2.53E-11 |
| 44Ar20 | Renal_arcuate_R | 1.00 | 0.85 | 1800 | 0.27 | 3.22E+10 | 2.53E-11 |

# 0D renal microcirculation model

The following three steps were adopted to calculate the PVR in the downstream of the arcuate artery:

1. Based on the circuit analogy of the nephron illustrated in Fig. S3g, the vascular resistance of a nephron was calculated based on nodal analysis using Kirchhoff’s circuit law. The detailed calculation was provided from Eq. 1 to 12.
2. Based on the parallel circuit configuration between all nephrons connected to an interlobular artery, the total vascular resistance of the nephrons was calculated using Eq. 14.
3. Based on the circuit configuration downstream of the arcuate artery illustrated in Fig. S3f, the PVR was calculated by combining the parallel arrangement of interlobular arteries with the series configuration of nephrons connected to each interlobular artery. The detailed calculation was provided in Eq. 15.

The vascular resistance of a nephron was determined by two steps. Firstly, nodal analysis was applied to the circuit representation illustrated in Fig. S3g, in conjunction with Eq. 1 to 12, we calculated the vascular resistance downstream of the afferent arteriole. Secondly, the vascular resistance of a nephron was calculated by summing the resistance of the afferent arteriole and the downstream components, based on their series configuration, using Eq. 14. The steps involved in the nodal analysis were presented below.

(1) Apply nodal analysis to nodes b, c and d, as shown in Fig. S3g,

$$\begin{aligned} \begin{matrix} Q_{toal}=Q_{1}+Q_{2} \\ Q_{2}=Q_{3}+Q_{5} \\ Q_{4}=Q_{1}+Q_{3} \end{matrix}\#\left( 1 \right) \end{aligned}$$

(2) Apply the constitutive laws of resistive elements, $Q=\frac{\Delta P}{R}$, where both the outlet pressures of $P_{e}$ and $P_{f}$ were set to 0 Pa,

$$\begin{aligned} \begin{matrix} Q_{1}=\frac{P_{b}-P_{c}}{R_{3}+R_{7}} \\ Q_{2}=\frac{P_{b}-P_{d}}{R_{4}+R_{5}} \\ \begin{matrix} Q_{3}=\frac{P_{b}-P_{d}+P_{o}}{R_{6}} \\ \begin{matrix} Q_{4}=\frac{P_{c}}{R_{8}} \\ Q_{5}=\frac{P_{d}}{R_{9}} \end{matrix} \end{matrix} \end{matrix}\#\left( 2 \right) \end{aligned}$$

(3) Substitute Eq.2 into Eq.1,

$$\begin{aligned} \begin{matrix} Q_{toal}=Q_{1}+Q_{2}=\frac{P_{b}-P_{c}}{R_{3}+R_{7}}+\frac{P_{b}-P_{d}}{R_{4}+R_{5}} \\ Q_{2}=Q_{3}+Q_{5}=\frac{P_{b}-P_{d}+P_{o}}{R_{6}}+\frac{P_{d}}{R_{9}} \\ Q_{4}=Q_{1}+Q_{3}=\frac{P_{b}-P_{c}}{R_{3}+R_{7}}+\frac{P_{b}-P_{d}+P_{o}}{R_{6}} \end{matrix}\#\left( 3 \right) \end{aligned}$$

(4) Write pressure drops across the resistive elements in terms of nodal pressures $P_{c}$ and $P_{e}$,

$$\begin{aligned} \begin{matrix} P_{c}=P_{b}-Q_{1}\left( R_{3}+R_{7} \right) \\ P_{d}=P_{b}-Q_{2}\left( R_{4}+R_{5} \right) \end{matrix}\#\left( 4 \right) \end{aligned}$$

(5) Substitute $P_{c}$, and $P_{d}$ from Eq.4 into the expression for $Q_{4}$ and $Q_{5}$ in Eq.2, and then substitute $Q_{4}$ and $Q_{5}$ using the relation provided in Eq.3,

$$\begin{aligned} \begin{matrix} \frac{P_{b}-Q_{1}\left( R_{3}+R_{7} \right)}{R_{8}}=Q_{1}+Q_{3} \\ \frac{P_{b}-Q_{2}\left( R_{4}+R_{5} \right)}{R_{9}}=Q_{2}-Q_{3} \end{matrix}\#\left( 5 \right) \end{aligned}$$

(6) Substitute Eq.4 into $Q_{3}$ in Eq.2,

$$\begin{aligned} Q_{3}=\frac{Q_{1}\left( R_{3}+R_{7} \right)-Q_{2}\left( R_{4}+R_{5} \right)+P_{o}}{R_{6}}\#\left( 6 \right) \end{aligned}$$

(7) Substitute Eq.6 into Eq.5,

$$\begin{aligned} \begin{matrix} \frac{P_{b}-Q_{1}\left( R_{3}+R_{7} \right)}{R_{8}}=Q_{1}+\frac{Q_{1}\left( R_{3}+R_{7} \right)-Q_{2}\left( R_{4}+R_{5} \right)+P_{o}}{R_{6}} \\ \frac{P_{b}-Q_{2}\left( R_{4}+R_{5} \right)}{R_{9}}=Q_{2}-\frac{Q_{1}\left( R_{3}+R_{7} \right)-Q_{2}\left( R_{4}+R_{5} \right)+P_{o}}{R_{6}} \end{matrix}\#\left( 7 \right) \end{aligned}$$

(8) Rewrite Eq.7, as $R_{3}$ and $R_{7}$, $R_{4}$ and $R_{5}$, are connected in series. For the convenience of subsequent derivations, this study defined $R_{37}=R_{3}+R_{7}$ and $R_{45}=R_{4}+R_{5}$,

$$\begin{aligned} \begin{matrix} \frac{P_{b}-Q_{1}R_{37}}{R_{8}}=Q_{1}+\frac{Q_{1}R_{37}-Q_{2}R_{45}+P_{o}}{R_{6}} \\ \frac{P_{b}-Q_{2}R_{45}}{R_{9}}=Q_{2}-\frac{Q_{1}R_{37}-Q_{2}R_{45}+P_{o}}{R_{6}} \end{matrix}\#\left( 8 \right) \end{aligned}$$

(9) Rearrange Eq.8,

$$\begin{aligned} \begin{matrix} Q_{1}\left( R_{37}R_{6}+R_{6}R_{8}+R_{37}R_{8} \right)-Q_{2}\left( R_{45}R_{8} \right)=P_{o}R_{6}-P_{o}R_{8} \\ -Q_{1}\left( R_{37}R_{9} \right)+Q_{2}\left( R_{45}R_{6}+R_{6}R_{9}+R_{45}R_{9} \right)=P_{o}R_{6}+P_{o}R_{8} \end{matrix}\#\left( 9 \right) \end{aligned}$$

(10) For notational simplicity and to facilitate subsequent analysis, the coefficients in Eq. 9 were defined as follows,

$$\begin{aligned} \begin{matrix} A=R_{37}R_{6}+R_{6}R_{8}+R_{37}R_{8} \\ B=-R_{45}R_{8} \\ \begin{matrix} C=P_{o}R_{6}-P_{o}R_{8} \\ D={-R}_{37}R_{9} \\ \begin{matrix} E=R_{45}R_{6}+R_{6}R_{9}+R_{45}R_{9} \\ F=P_{o}R_{6}+P_{o}R_{9} \end{matrix} \end{matrix} \end{matrix}\#\left( 10 \right) \end{aligned}$$

(11) In matrix form,

$$\begin{aligned} \left[ \begin{matrix} A & B \\ D & E \end{matrix} \right]\left[ \begin{matrix} Q_{1} \\ Q_{2} \end{matrix} \right]=\left[ \begin{matrix} C \\ F \end{matrix} \right]\#\left( 11 \right) \end{aligned}$$

(12) Solve for $Q_{1}$ and $Q_{2}$,

$$\begin{aligned} \begin{matrix} Q_{1}=\frac{CE-BF}{AE-BD} \\ Q_{2}=\frac{AF-CD}{AE-BD} \end{matrix}\#\left( 12 \right) \end{aligned}$$

(13) Solve for total vascular resistance ($R_{total}$) of a single nephron using the provided values for inlet and outlet pressures, osmotic pressure drops, and vascular resistances of individual components within the renal circulation,

$$\begin{aligned} R_{glomerulus}=R_{2}+\frac{P_{b}}{Q_{total}}=R_{2}+\frac{P_{b}\left( AE-BD \right)}{CE-BF+AF-CD}\#\left( 13 \right) \end{aligned}$$

$$\begin{matrix} P_{b}=7980 Pa \\ P_{o}=1995 Pa \\ \begin{matrix} R_{2}=8.71\times{10}^{13} Pa\cdot s\cdot m^{-3} \\ R_{3}=3.92\times{10}^{12} Pa\cdot s\cdot m^{-3} \\ \begin{matrix} R_{4}=3.35\times{10}^{14} Pa\cdot s\cdot m^{-3} \\ R_{5}=1.27\times{10}^{15} Pa\cdot s\cdot m^{-3} \\ \begin{matrix} R_{6}=3.31\times{10}^{14} Pa\cdot s\cdot m^{-3} \\ R_{7}=1.52\times{10}^{14} Pa\cdot s\cdot m^{-3} \\ \begin{matrix} R_{8}=3.60\times{10}^{13} Pa\cdot s\cdot m^{-3} \\ R_{9}=7.96\times{10}^{15} Pa\cdot s\cdot m^{-3} \end{matrix} \end{matrix} \end{matrix} \end{matrix} \end{matrix}$$

Based on the second and third assumptions, the total vascular resistance associated with all nephrons connected to the distal end of one interlobular renal artery was calculated by Eq. 14.

$$\begin{aligned} \begin{matrix} \frac{1}{R_{glomeruli}}=\frac{1}{R_{1}}+\frac{1}{R_{2}}+\frac{1}{R_{3}}+\ldots+\frac{1}{R_{60}} \\ R_{1}=R_{2}=\ldots=R_{60}=R_{glomerulus} \\ R_{glomeruli}=\frac{R_{glomerulus}}{60} \end{matrix}\#\left( 14 \right) \end{aligned}$$

Finally, the total vascular resistance associated with all interlobular renal arteries and nephrons connected to the distal end of one arcuate renal artery was calculated through Eq. 15.

$$\begin{aligned} R_{PVR}=\frac{R_{interlobular}+R_{glomeruli}}{N_{interlobular}}\#\left( 15 \right) \end{aligned}$$

The PVC of the interlobular artery was calculated by Eq. 16. In this context, $C_{t}$ represented the PVC assigned to the outlet of the main renal artery. The $C_{i}$ was calculated step by step for the segmental, interlobar, and arcuate arteries, with the final value for the arcuate arteries used to represent the PVC of the interlobular arteries.

$$\begin{aligned} \frac{C_{i}}{C_{t}}=\frac{A_{i}}{A_{t}}\#\left( 16 \right) \end{aligned}$$

- $C_{i}$ is the PVC of the individual outlet
- $C_{t}$ is the total PVC of the outlets
- $A_{i}$ is the cross-sectional area of the individual outlet
- $A_{t}$ is the total cross-sectional area of the outlets

From the above derivation, the PVR in renal circulation is 2.80×10^10^ Pa·s/m^3^ and PVC in renal circulation is 2.10×10^-11^ m^3^·Pa^-1^. According to run a preliminary simulation compared with *in vivo* literature data, the derived PVR is used to represent male, and the PVC is used to represent female. The corresponding values for non-sex-specific baseline model (see sex-specific model) is reported in the Table S2.


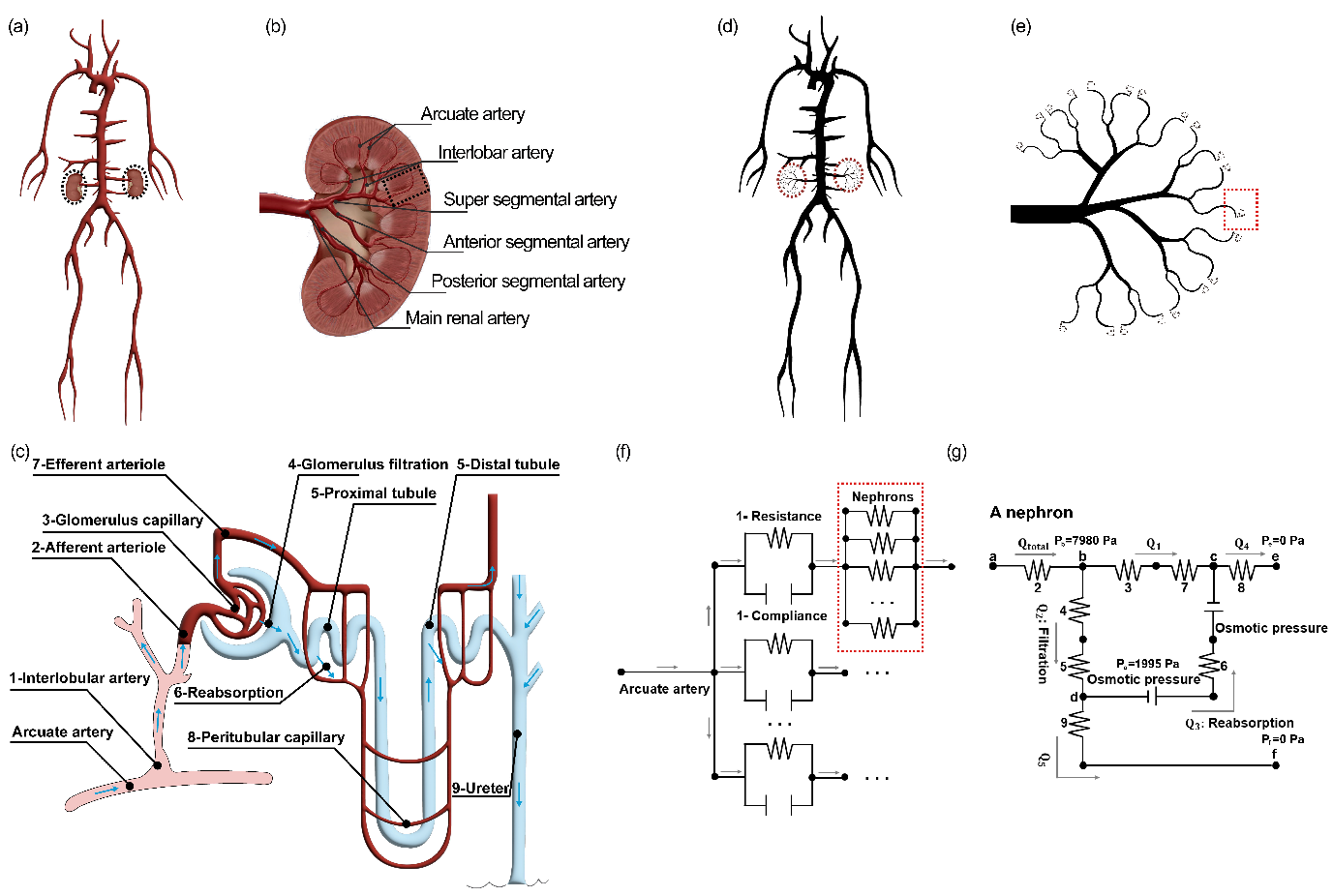


Fig. S3 Illustration of the systemic whole-body circulation and renal circulation, including both anatomical structures and model representations

# Computation of glomerular filtration rate

In this study, it was assumed that all glomeruli simultaneously filter the blood entering the renal microcirculation and that each glomerulus exhibited an identical filtration rate. In this context, $Q_{total}$ denoted the total volumetric blood flow entering a single glomerulus through its afferent arteriole, while $Q_{2}$ represented the volumetric flow rate of blood filtered by the glomerulus, as shown in Fig. S3g. The ratio between and $Q_{total}$ defined the filtration rate, which was referred to as the blood filtration fraction in this study.

According to the principle of flow conservation, the volumetric flow rate of blood entering the main renal artery is equal to the volumetric flow rate reaching the glomeruli and subsequently undergoing filtration. In this study, Eqs. 1, 12, and 17 were applied to calculate the GFR of virtual individuals, and the blood filtration fraction was calculated to be 10%, 12%, and 11% for healthy, DKD, and HKD individuals, respectively. For virtual patients with DM+HTN and DM+HTN+HKD, a 5% constriction of the afferent and efferent arterioles was considered when calculating GFR.

$$\begin{aligned} glomerular filtration rate \left( GFR \right)=Q_{mean}\cdot\frac{Q_{2}}{Q_{total}}\#\left( 17 \right) \end{aligned}$$

- $Q_{mean}$ is the mean blood volumetric flow rate throughout a complete cardiac cycle in the main renal artery
- $Q_{2}$ is the blood volumetric flow rate filtered by the glomerulus
- $Q_{total}$ is the total blood volumetric flow rate entering the glomerulus

# Age-specific model

Table S3 presents the age-specific model to parameterize the ageing process.

Table S3 Distribution of scaling parameters for the ageing model

| Parameters  Mean (SD) | Age groups [year old] | | | | | |
| --- | --- | --- | --- | --- | --- | --- |
|  | 20-29 | 30-39 | 40-49 | 50-59 | 60-69 | 70-79 |
| Cardiac output | 1.00 (0.23) | 0.95 (0.22) | 0.90 (0.22) | 0.87 (0.22) | 0.80 (0.22) | 0.75 (0.22) |
| Length | 1.00 (0.13) | 1.08 (0.14) | 1.16 (0.16) | 1.22 (0.17) | 1.32 (0.18) | 1.40 (0.19) |
| Radius | 1.00 (0.06) | 1.03 (0.06) | 1.04 (0.07) | 1.07 (0.07) | 1.15 (0.10) | 1.18 (0.11) |
| Stiffness | 1.00 (0.16) | 1.10 (0.18) | 1.23 (0.16) | 1.32 (0.25) | 1.60 (0.35) | 2.00 (0.45) |
| Thickness | 1.00 (0.18) | 1.10 (0.20) | 1.12 (0.22) | 1.25 (0.22) | 1.51 (0.26) | 1.75 (0.28) |
| PVR | 1.00 (0.22) | 1.06 (0.24) | 1.15 (0.25) | 1.25 (0.27) | 1.37 (0.28) | 1.46 (0.29) |
| PVC | 1.00 (0.27) | 0.89 (0.24) | 0.77 (0.21) | 0.65 (0.18) | 0.58 (0.15) | 0.50 (0.11) |
| Number  Male  Female | 503  471 | 457  480 | 511  543 | 514  600 | 500  626 | 432  551 |

# AUC in different classifiers

Fig. S4 presents AUCs derived from ROC curves for logistic regression, support vector machine, decision tree, and random forest classifiers distinguishing virtual male patients with DKD and HKD in the 50-59 age group.


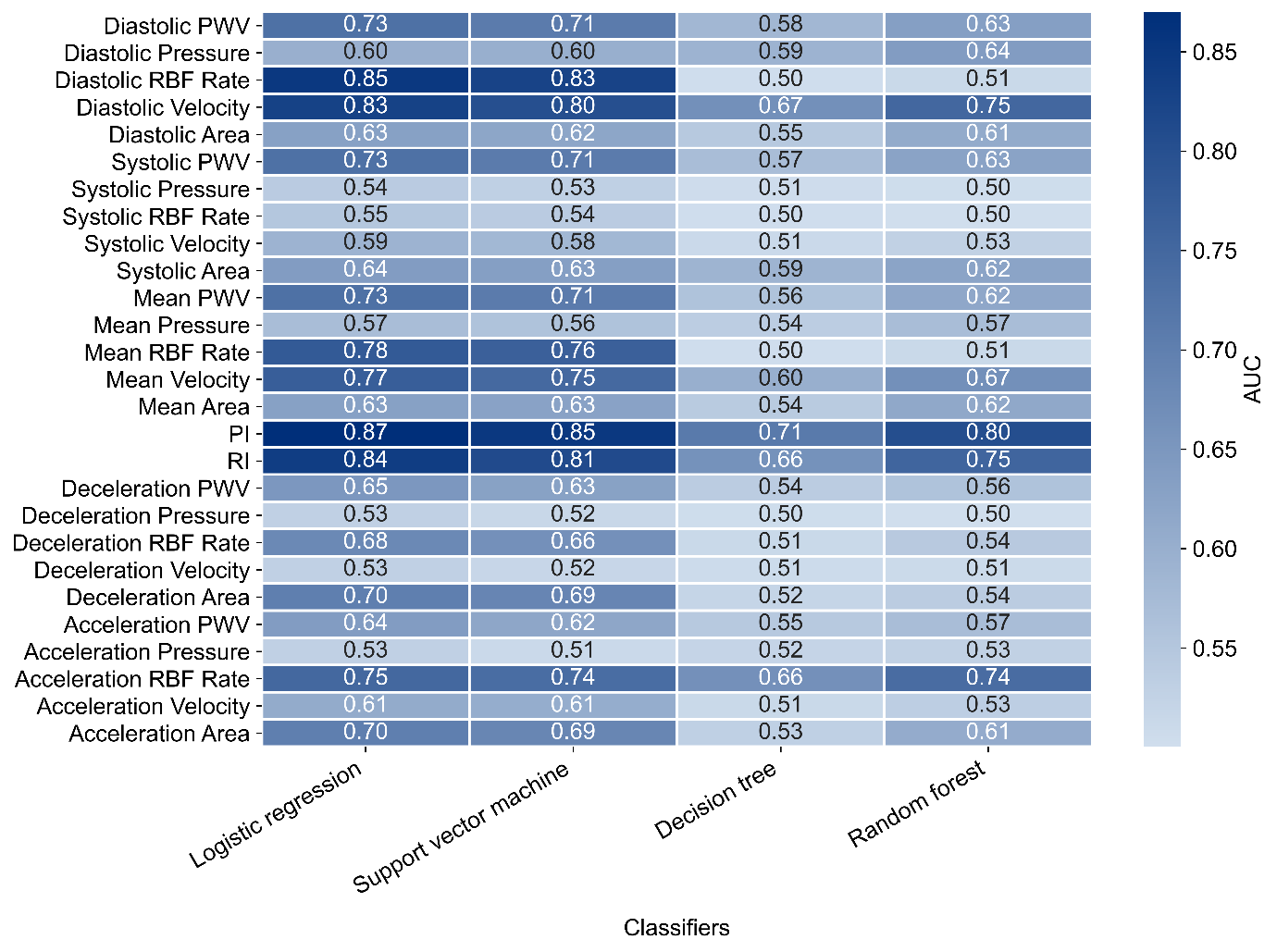


Fig. S4 AUC for virtual male patients with DKD and HKD within the 50-59 yo age group, classified by logistic regression, support vector machine, decision tree, and random forest

# AUC in sex-specific virtual patients

Fig. S5 presents absolute difference in AUC from ROC curves using logistic regression models, comparing virtual male and female patients with DKD and HKD in the 50-59 age group.


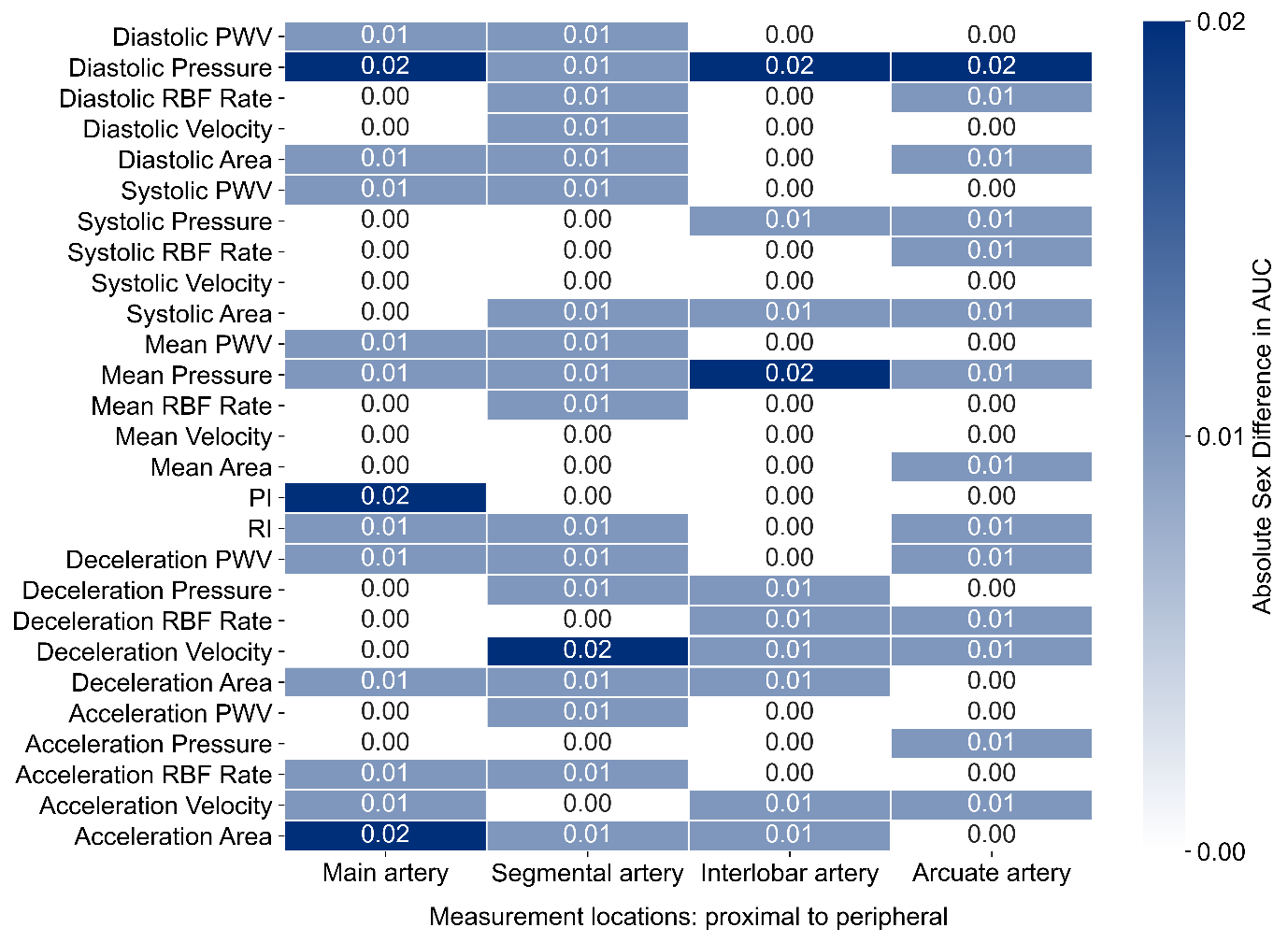


Fig. S5 Absolute difference in AUC between virtual male and female patients with DKD and HKD within the 50-59 yo age group

# AUC in age-specific virtual patients

Fig. S6 presents AUCs derived from ROC curves using logistic regression models in virtual male patients with DKD and HKD across six different age groups.


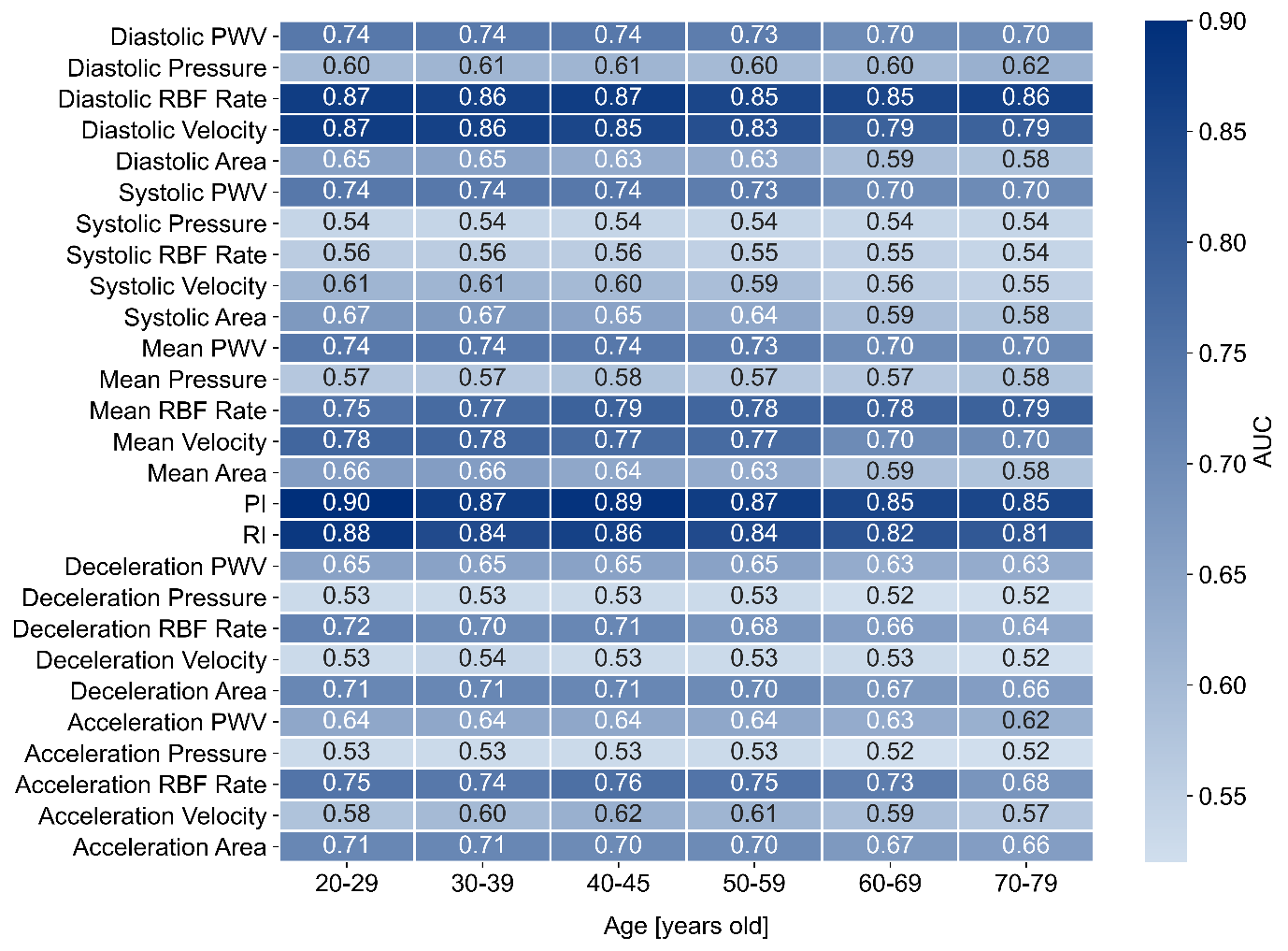


Fig. S6 AUC for virtual male patients with DKD and HKD across six different age groups

# AUC in age-specific virtual patients

Table S3 presents the best cutoff for each biomarker, derived from ROC curves fitted with logistic regression models.

Table S3 Best cutoff for each biomarker in male virtual patients with DKD and HKD within the 50-59 yo age group. Unit: PWV and velocity, cm/s; RBF rate, ml/min; area, cm^2^

| Biomarker | Best cutoff | Sensitivity | Specificity |
| --- | --- | --- | --- |
| Diastolic PWV | 11.68 | 0.71 | 0.65 |
| Diastolic Pressure | 81.90 | 0.55 | 0.62 |
| Diastolic RBF Rate | 180.00 | 0.78 | 0.78 |
| Diastolic Velocity | 9.00 | 0.79 | 0.74 |
| Diastolic Area | 0.35 | 0.65 | 0.57 |
| Systolic PWV | 11.84 | 0.71 | 0.65 |
| Systolic Pressure | 141.59 | 0.61 | 0.50 |
| Systolic RBF Rate | 780.00 | 0.64 | 0.48 |
| Systolic Velocity | 34.00 | 0.62 | 0.56 |
| Systolic Area | 0.37 | 0.67 | 0.57 |
| Mean PWV | 11.76 | 0.71 | 0.65 |
| Mean Pressure | 109.31 | 0.64 | 0.50 |
| Mean RBF Rate | 360.00 | 0.67 | 0.74 |
| Mean Velocity | 17.00 | 0.73 | 0.70 |
| Mean Area | 0.36 | 0.64 | 0.58 |
| PI | 1.41 | 0.80 | 0.80 |
| RI | 0.72 | 0.77 | 0.77 |
| Deceleration PWV | 0.21 | 0.63 | 0.62 |
| Deceleration Pressure | 81.49 | 0.55 | 0.54 |
| Deceleration RBF Rate | 720.00 | 0.66 | 0.63 |
| Deceleration Velocity | 31.00 | 0.52 | 0.57 |
| Deceleration Area | 0.03 | 0.74 | 0.58 |
| Acceleration PWV | 0.56 | 0.63 | 0.61 |
| Acceleration Pressure | 220.48 | 0.55 | 0.54 |
| Acceleration RBF Rate | 2400.00 | 0.73 | 0.66 |
| Acceleration Velocity | 105.00 | 0.57 | 0.63 |
| Acceleration Area | 0.07 | 0.73 | 0.59 |
